# Quantitative Structure–Activity Relationships for Structurally Diverse Chemotypes Having Anti-*Trypanosoma cruzi* Activity

**DOI:** 10.3390/ijms20112801

**Published:** 2019-06-08

**Authors:** Anacleto S. de Souza, Leonardo L. G. Ferreira, Aldo S. de Oliveira, Adriano D. Andricopulo

**Affiliations:** 1 Laboratory of Computational and Medicinal Chemistry, Center for Research and Innovation in Biodiversity and Drug Discovery, Physics Institute of Sao Carlos, University of Sao Paulo, Sao Carlos-SP 13563-120, Brazil; anacletosilvadesouza@usp.br (A.S.d.S.); leonardo@ifsc.usp.br (L.L.G.F.); aldo.sena@ufsc.br (A.S.d.O.); 2 Department of Exact Sciences and Education, Blumenal Center, Federal University of Santa Catarina, Blumenau 89036-256, Brazil

**Keywords:** artificial neural networks, machine learning, Chagas’ disease, QSAR, molecular modeling

## Abstract

Small-molecule compounds that have promising activity against macromolecular targets from *Trypanosoma cruzi* occasionally fail when tested in whole-cell phenotypic assays. This outcome can be attributed to many factors, including inadequate physicochemical and pharmacokinetic properties. Unsuitable physicochemical profiles usually result in molecules with a poor ability to cross cell membranes. Quantitative structure-activity relationship (QSAR) analysis is a valuable approach to the investigation of how physicochemical characteristics affect biological activity. In this study, artificial neural networks (ANNs) and kernel-based partial least squares regression (KPLS) were developed using anti-*T. cruzi* activity data for broadly diverse chemotypes. The models exhibited a good predictive ability for the test set compounds, yielding *q*^2^ values of 0.81 and 0.84 for the ANN and KPLS models, respectively. The results of this investigation highlighted privileged molecular scaffolds and the optimum physicochemical space associated with high anti-*T. cruzi* activity, which provided important guidelines for the design of novel trypanocidal agents having drug-like properties.

## 1. Introduction

**Figure 1:**
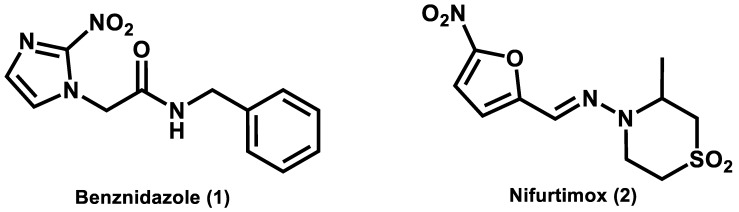
Structures of benznidazole and nifurtimox, which are the only two available drugs for the chemotherapy of Chagas’ disease.

Chagas’ disease, which is a neglected tropical disease (as defined by the World Health Organization, WHO) caused by the protozoan *Trypanosoma cruzi*, is the leading cause of heart failure in Latin America, where it is endemic [1]. According to the WHO, the disease affects 8 million people worldwide and causes 10,000 deaths every year. Moreover, more than 25 million people live in vulnerable areas under the risk of infection [2]. Current chemotherapy for Chagas’ disease is limited to nifurtimox and benznidazole, which are two obsolete drugs identified in 1965 and 1971, respectively (Figure 1). These nitroheterocyclic compounds cause several adverse effects, such as weight loss, neurological damage, anorexia, dermatitis, depression, nausea, and gastrointestinal problems [3,4]. Furthermore, they lack effectiveness in the chronic phase of the disease. Given these drawbacks, novel, effective, and safe drugs for Chagas’ disease are urgently needed [5].

*T. cruzi* parasites interconvert into different morphological phases during their life cycle as they circulate between the insect host (the Triatomine bugs *Triatoma infestans*, *Rhodnius prolixus*, and *Triatoma dimidiata*) and the human host. Replicative epimastigotes and infective metacyclic trypomastigotes develop in Triatomine bugs, whereas replicative intracellular amastigotes and non-replicative bloodstream trypomastigotes develop in humans [6]. Intracellular amastigotes, which are found in tissues such as cardiac muscles and the digestive system, are the clinically relevant form of the parasite, and thus are the targets of antichagasic agents [6,7]. Occasionally, compounds that are active against isolated macromolecular targets lose their activity when tested in whole-cell phenotypic assays [8,9,10,11]. This activity loss can stem from inappropriate physicochemical properties, which play a key role in the ability of compounds to permeate biological membranes and reach their molecular targets [12,13]. In this context, drug discovery players have unprecedentedly relied on chemoinformatics to better understand the relationships between structure, physicochemical properties, and biological activity [14,15,16]. Quantitative structure-activity relationships (QSAR) have played a major role in this field [17,18,19]. In this study, we developed artificial neural networks (ANNs) and kernel-based partial least squares models (KPLS) aimed at investigating the molecular events underlying the activity of structurally diverse trypanocidal agents [20,21]. The outcome of these models was used to generate a focused fragment collection and physicochemical heat maps, which provide insights into privileged chemotypes and optimum physicochemical property spaces associated with enhanced trypanocidal activity.

ANNs are aimed to mimic biological neural networks and their processing units, the neurons, are composed of dendrites, a cell body, and axons. All input values (the dendrites) are summed and then are assigned to a learning function (the cell body). The input values are the independent variables and the output values are the dependent variables. The signal (axon) can be propagated or inhibited if the value returned by the activation function is above or below a predetermined threshold, respectively [22]. The multi-layer back-propagation algorithm was used in the ANN models. In particular, the back-propagation method uses the forward and backward steps [23]. First, weights are determined, and the biological activity value is predicted for a compound. The error between experimental and predicted values provides support for adjusting the input weight in the first intermediate layer. The main limitation of the algorithm is the convergence of the network due to low and high values in the learning rate. To reduce this limitation, the term momentum ensures that the learning rate is stabilized.

The fingerprint descriptors in the KPLS models are calculated from the smiles representation of each structure in the dataset [24]. These descriptors can be classified as linear, dendritic, radial, and molprint2D [25]. These four descriptors allow the visualization of atomic contribution maps, which depict the contribution of each atom to the dependent variable. The linear fingerprint descriptor uses the information from the linear fragments and ring closure to convert the structures into binary sequences. The dendritic fingerprint includes branched parts of the molecule during the generation of the binary sequence. Also referred to as extended connectivity fingerprints, the radial fingerprint identifies all heavy atoms and encodes the compounds by assigning fragments that emerge radially from each atom. Finally, molprint2D is similar to the radial fingerprint and encodes the heavy atom environments by identifying the atom types positioned at different topological distances.

## 2. Results

### 2.1. Chemical and Biological Landscape

**Figure 2:**
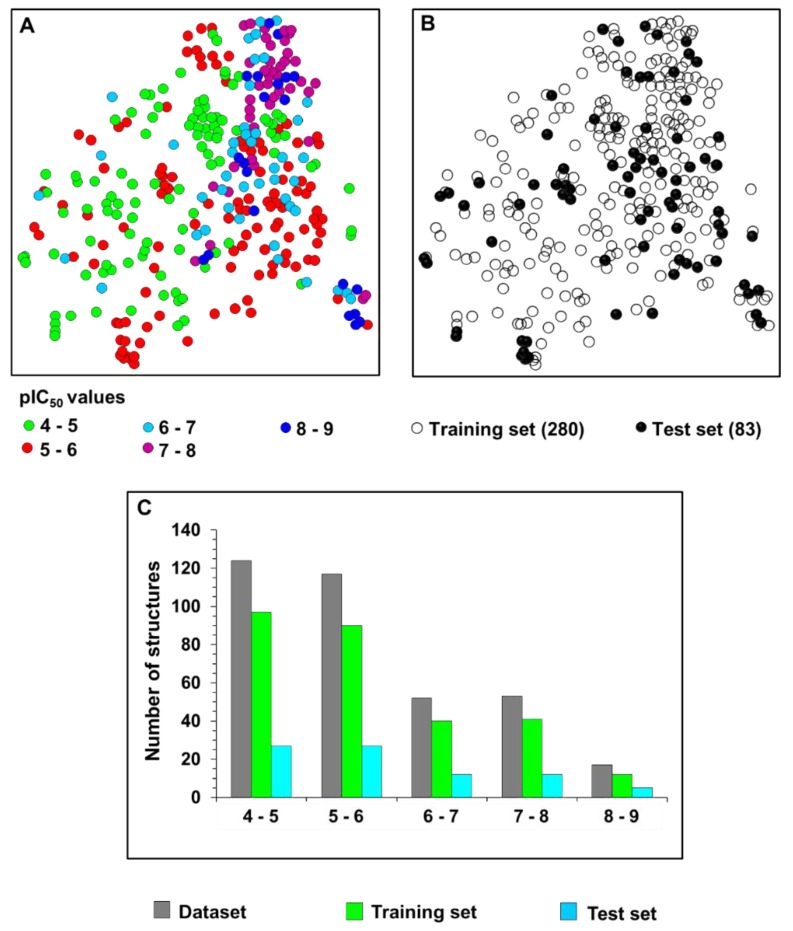
Structural and activity landscape of the dataset. (**A**) Structure similarity map for the entire dataset composed of 363 compounds, which shows its broad chemical diversity and activity range. (**B**) Structure similarity map highlighting the training (open circles) and test set (solid circles) compounds. (**C**) Activity distribution for the whole dataset and for the training and test sets.

The dataset used to construct the ANN and KPLS models was selected from the literature and includes 363 structurally diverse compounds [26,27,28,29,30,31,32,33,34,35,36,37,38,39,40,41,42,43,44,45,46,47,48,49,50,51,52,53,54,55,56,57,58,59,60,61,62,63,64]. The trypanocidal activity of these molecules is expressed as the concentration of the compound that inhibits 50% the growth of *T. cruzi* in phenotypic assays (IC_50_). The IC_50_ values range from 2 nM to 97.97 µM (a 48,985-fold activity range) and were converted into pIC_50_ values (−log IC_50_) prior to the QSAR modeling. This wide activity interval follows the broad chemical diversity enclosed in the dataset. The structural and activity landscape covered by the 363 trypanocidal agents is illustrated in Figure 2. In the structure similarity map, the distance among the points is inversely proportional to the structural similarity, and the colors represent different activity ranges (Figure 2A). Based on this map, training and test sets were selected to construct the models. Structurally distinct chemotypes enclosing a wide spectrum of pIC_50_ values were included in both the training (280 compounds) and test sets (83 compounds), as depicted in Figure 2B,C.

### 2.2. Artificial Neural Networks

**Figure 3:**
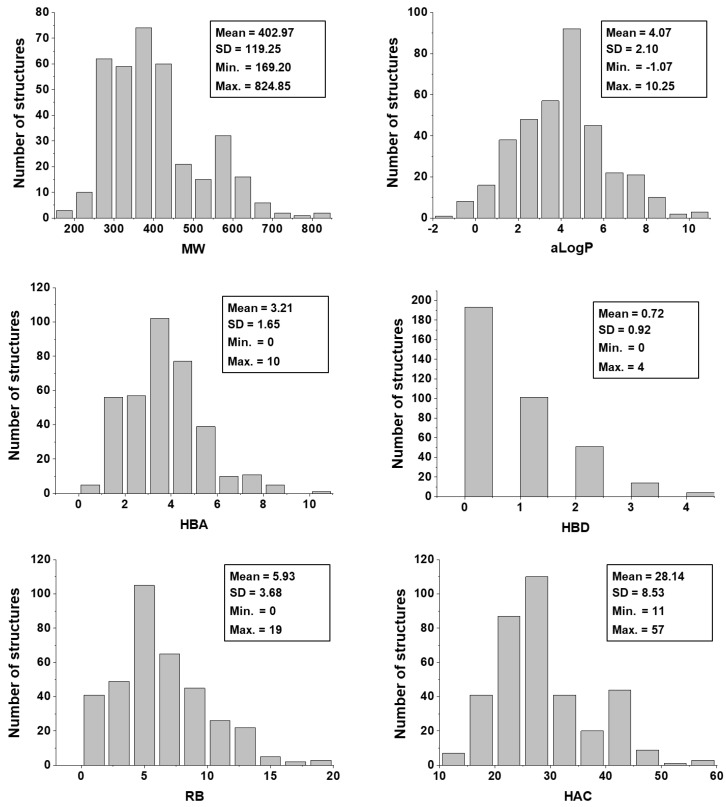
Distribution of physicochemical properties for the dataset. Note: MW = molecular weight; *a*LogP = logarithm of the octanol-water partition coefficient; HBA = hydrogen bond acceptors; HBD = hydrogen bond donors; RB = number of rotatable bonds; HAC = heavy atom count; SD = standard deviation.

**Figure 4:**
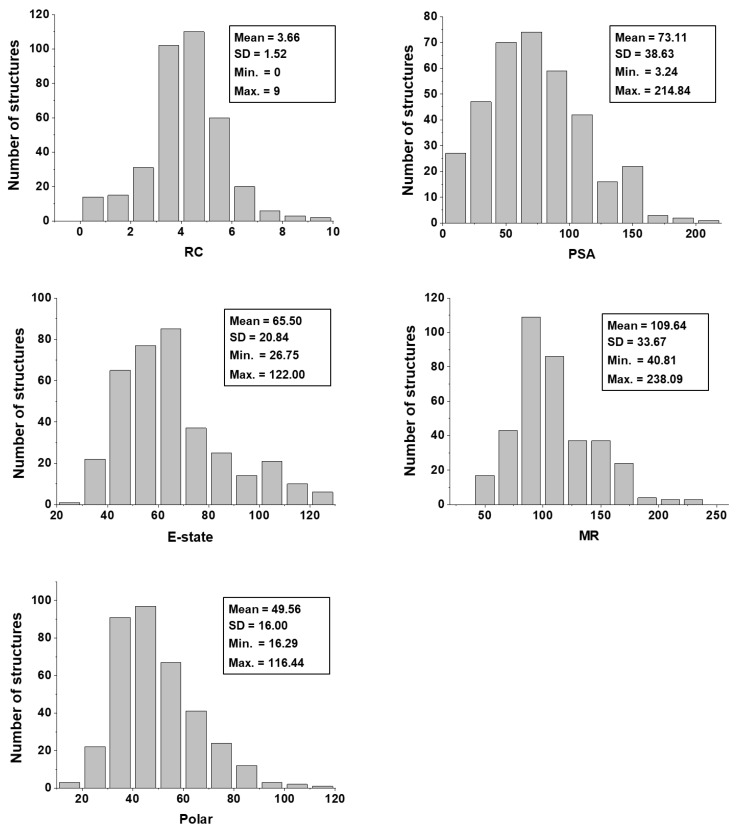
Physicochemical characterization of the dataset. Note: RC = ring count; PSA = polar surface area; E-state = electrotopological state; MR = molar refractivity; Polar = molecular polarizability; SD = standard deviation.

Eleven physicochemical properties were used as molecular descriptors to build the ANNs through which the trypanocidal activity of the dataset compounds were predicted. Hence, prior to running the ANN analyses, the dataset was characterized with respect to its physicochemical profile. Figure 3 shows the distribution of the dataset regarding the following physicochemical descriptors: molecular weight (MW), octanol-water partition coefficient (*a*LogP), hydrogen bond acceptors (HBA), hydrogen bond donors (HBD), number of rotatable bonds (RB), and heavy atom count (HAC). Figure 4, in turn, shows the physicochemical profile of the dataset regarding ring count (RC), polar surface area (PSA), electrotopological state (E-state), molar refractivity (MR), and molecular polarizability (Polar).

The calculated physicochemical descriptors for 280 training set compounds were used as inputs to build backpropagation ANNs. The predictive power of the models was additionally evaluated by using 83 test set molecules. Table 1 shows the performance of the ANNs as a function of the learning rate (LR). The score value was used as the leading parameter to evaluate the performance of the models. The top-scoring model, which was derived with an LR of 0.1, presented a score of 0.80. As the score is derived from the correlation coefficients, this model had good performance regarding these indicators for both the training and test sets (*r*² = 0.79 and *q*² = 0.85). The *r*² value indicates the internal statistical robustness of the models considering the training set only. Otherwise, the *q*² value indicates the external predictive power, since it considers the performance of the models in predicting the dependent variables of molecules that were not used to train the algorithm. The score value represents the general performance of the models, considering both its internal and external predictive power.

Next, the momentum parameter (MP) of the backpropagation algorithm was varied for the top-scoring model of Table 1. This procedure was conducted to search for the best convergence criterion and prevent the ANNs from converging to a local minimum. MP was varied from 0.1 to 0.4 in steps of 0.1, and the resulting models are shown in Table 2. As seen, the variation of MP had no influence on the overall quality of the ANNs, as demonstrated by the resulting scores. Hence, the lowest RMSE values (0.82 and 0.75 for the training and test sets, respectively) were considered to select the model having an MP of 0.2 and a score of 0.80 for further optimization. Importantly, this model maintained good correlation coefficients for both the training (*r*² = 0.79) and test sets (*q*² = 0.85).

Finally, we varied the number of neurons in the hidden layer from one to 10, while maintaining the optimal values of LR (0.1) and MP (0.2). The best results were produced when seven neurons were added to the hidden layer, as shown in Table 3. A subtle improvement in the score (0.81) was observed in this model compared to the ANN containing the default number of six neurons in the hidden layer.

**Figure 5:**
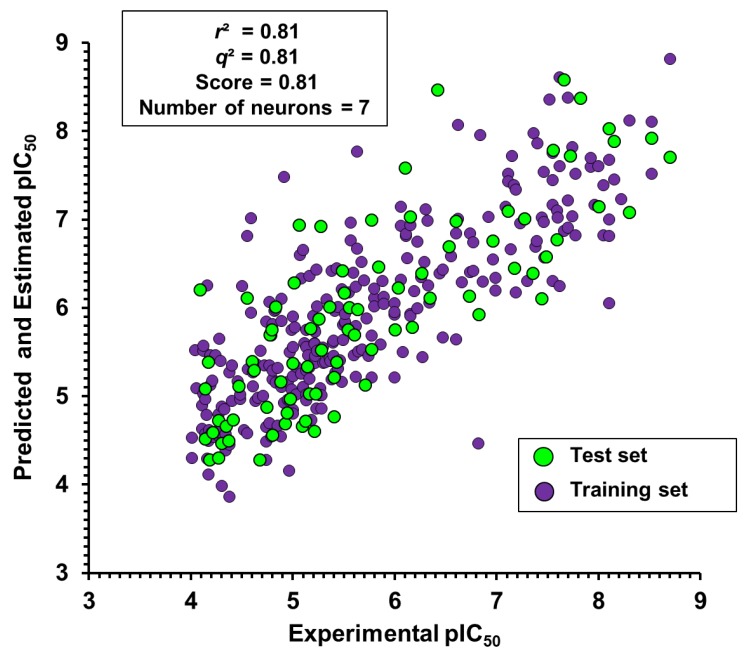
Experimental versus ANN-predicted values of trypanocidal activity (pIC_50_).

The statistical indicators shown in Table 3, particularly the correlation coefficient for the test compounds (*q*² = 0.81), show the ability of the best ANN model to predict the trypanocidal activity of novel and structurally diverse compounds. The good agreement between the experimental and predicted pIC_50_ values exhibited by this model is graphically illustrated in Figure 5 for the training and test set compounds.

#### Impact of the Physicochemical Properties on the Trypanocidal Activity

Given the high predictive power of the best ANN (seven neurons in the hidden layer, Table 3), we investigated the role played by each physicochemical descriptor on the trypanocidal activity of the dataset compounds. Each molecular descriptor works as an input applied to the ANN neurons. These descriptors are weighted by an activation function at each neuron, which is responsible for processing and transmitting the signal to the other neurons. These weights can be positive or negative. A positive weight attributed to a given physicochemical descriptor leads to a proportional increase in biological activity; that is, increasing the value of the weight increases the activity, and decreasing this value decreases the biological activity. Otherwise, negative weights lead to an inversely proportional neuron response, i.e., decreasing the weight value increases the biological activity, and increasing this value decreases the activity.

Table 4 shows the weights of each molecular descriptor at each hidden-layer neuron. Among the 11 descriptors, MW showed the greatest difference between the number of positively and negatively weighted neurons. Six neurons had their activation function positively weighted by MW; for most neurons, increments in this property resulted in increased trypanocidal activity. In the second position came *a*LogP, HBA, HBD, and MR, which positively weighted five out of the seven hidden-layer neurons. The reciprocal ratio was observed for RB, that is, five neurons produced negative values. HAC, RC, and Polar positively weighted four neurons, whereas PSA and E-state showed the inverse ratio, i.e., they negatively weighted four hidden-layer neurons.

### 2.3. Kernel-Based Partial Least Squares

**Figure 6:**
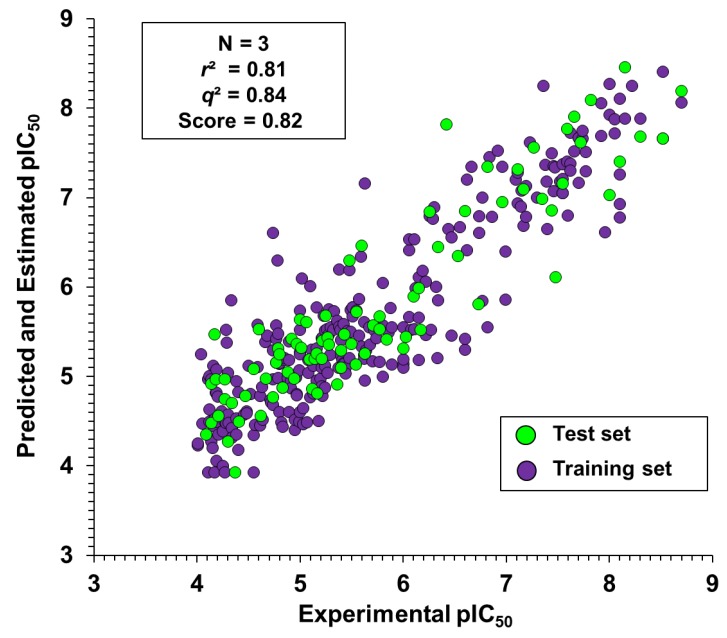
Experimental versus KPLS-predicted values of trypanocidal activity (pIC_50_).

Four molecular fingerprint types (dendritic, linear, molprint2D, and radial) were used as molecular descriptors to generate the 2D QSAR models. These descriptors were correlated with the trypanocidal activity of the dataset compounds using KPLS regression. The statistical indicators for the best models generated with each descriptor are presented in Table 5. All fingerprint types produced models with a similar prediction ability for the test set, with molprint2D performing slightly better (*q*^2^ = 0.84). For the training set, the best correlation coefficients were produced by models generated with dendritic and linear fingerprints (*r*^2^ = 0.89). The highest score, which is the result of the combination of *q*^2^ and *r*^2^, was produced by the molprint2D-model (score = 0.82). Figure 6 illustrates the good alignment between the experimental and predicted pIC_50_ values produced by the molprint2D-model. Considering these results, this model was selected to investigate how the structure of the dataset compounds correlate with the trypanocidal activity.

#### Contribution Maps

**Figure 7:**
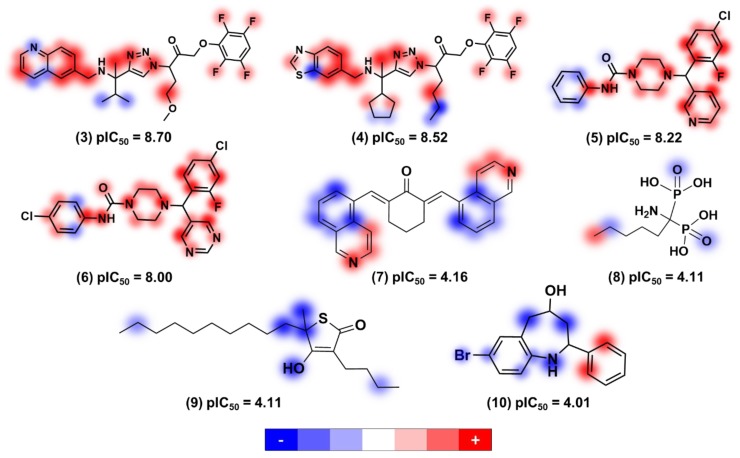
Contribution maps based on the molprint2D model (Table 5) for some dataset compounds located at both ends of the trypanocidal activity range.

KPLS models can be assessed for favorable and unfavorable structural characteristics through the generation of atomic contribution maps. Hence, the most relevant structural features for the trypanocidal activity were investigated by generating contribution maps based on the best KPLS model (molprint2D, Table 5). Positive, neutral, and negative contributions are depicted in red, white, and blue, respectively, and the color intensity shows the magnitude of the effect (Figure 7). Overall, heterocyclic aromatic rings contributed positively to activity. Aliphatic hydrocarbon chains showed negative or no influence for most compounds. Halogen substituents showed the full range of contributions—fluorine contributed positively, chlorine and iodine had no influence, and bromine contributed negatively. In general, hydroxyl groups were unfavorable, and piperazine rings were demonstrated to be favorable.

## 3. Physicochemical Profile of Favorable Fragments

**Figure 8:**
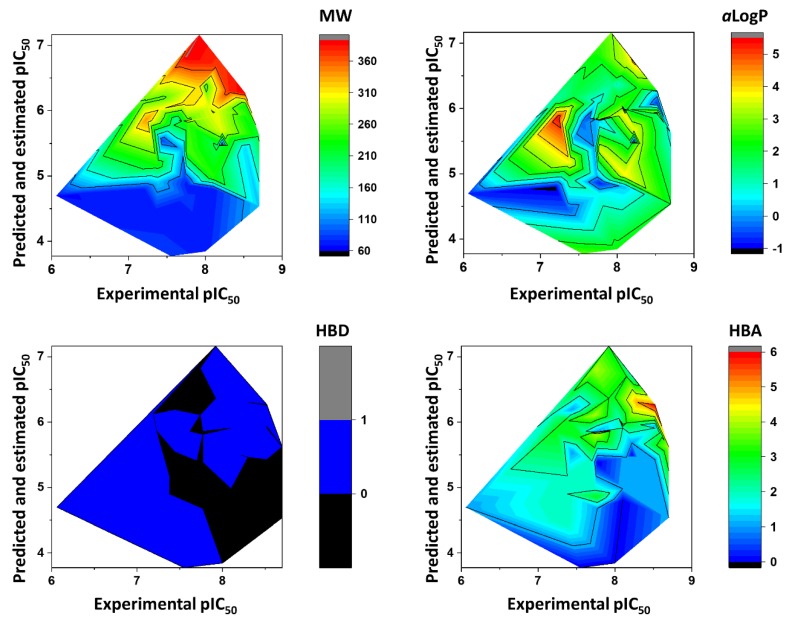
Heat maps showing the correlation between physicochemical properties and biological activity for molecular fragments extracted from the dataset. Note: MW = molecular weight; *a*LogP = octanol-water partition coefficient; HBD = hydrogen bond donors; HBA = hydrogen bond acceptors.

**Figure 9:**
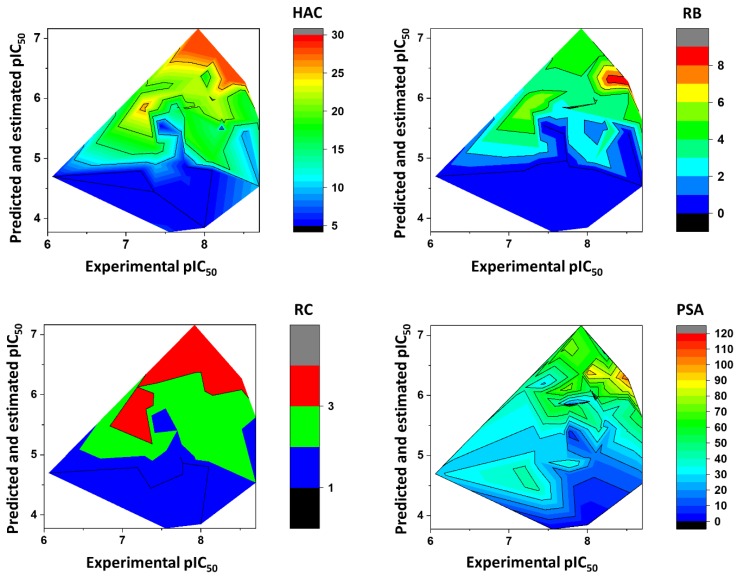
Heat maps showing the correlation between physicochemical properties and biological activity for molecular fragments extracted from the dataset. Note: HAC = heavy-atom count; RB = number of rotatable bonds; RC = ring count; PSA = polar surface area.

**Figure 10:**
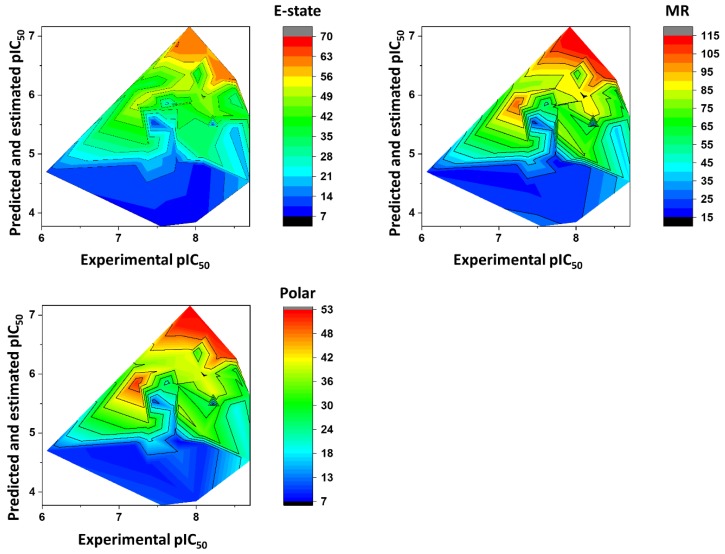
Heat maps showing the correlation between physicochemical properties and biological activity for molecular fragments extracted from the dataset. Note: E-state = electrotopological state; MR = molar refractivity; Polar = molecular polarizability.

Twenty-nine active compounds with pIC_50_ > 6 (see Table S1 for the structures) were selected to construct a collection of 50 fragments. Only molecular fragments that were predicted to enhance the trypanocidal activity (red areas of the contribution maps) were considered in this analysis. Next, physicochemical descriptors were calculated for these fragments and used as an input for the best ANN with the view of predicting their biological activity. Finally, the predicted activity values were correlated with each physicochemical property of the fragment collection. The outcome of this analysis is illustrated as heat maps (Figure 8, Figure 9 and Figure 10), which allowed us to identify a specific physicochemical space that favors trypanocidal activity. The heat maps also correlate the activity of the compounds from which the fragments were extracted and their physicochemical profile.

Figure 8 shows the heat maps for MW, *a*LogP, HBD, and HBA. Fragments with MW greater than 260 Da were predicted to be the most active (pIC_50_ > 6). For *a*LogP, the most active fragments had values predominantly between 2 and 3. Fragments with 0–1 HBD and 1–6 HBAs were predicted to have the highest pIC_50_ values. Figure 9 illustrates the heat maps for HAC, RB, RC, and PSA. As shown in the figure, fragments with HAC values greater than 20 had the highest pIC_50_ values. For RB, fragments with two to eight rotatable bonds were the most active. According to the heat maps, fragments with RC values from 2 to 3 were predicted to have the best anti-*T. cruzi* profile. Finally, fragments with polar surface area (PSA) predominantly between 50 and 80 Å^2^ had the highest pIC_50_ values. Figure 10 shows the heat maps for E-state, MR, and Polar. The ANN predicted the fragments with E-state values between 35 and 63 as being the most active. Fragments with MR ranging from 65 to 115 were predicted to have the highest pIC_50_ values. Finally, the Polar descriptor was demonstrated to have optimal values ranging from 30 to 53.

**Figure 11:**
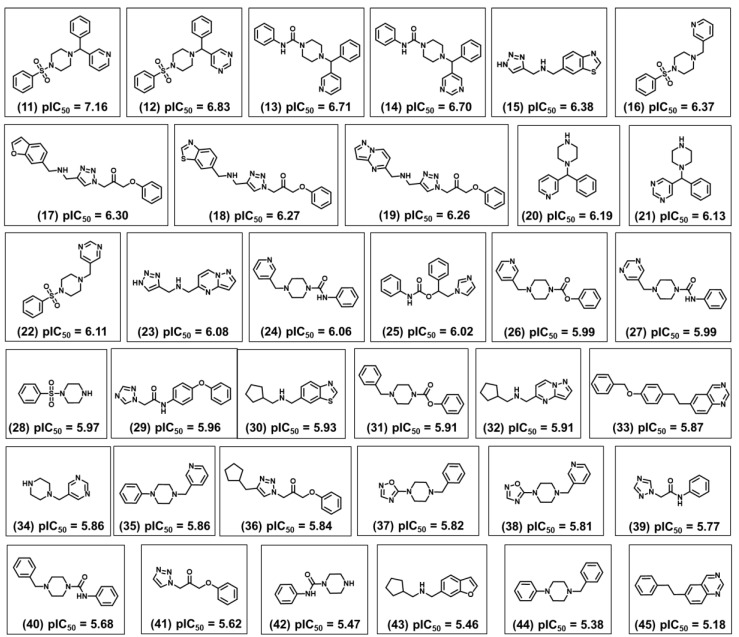
Fragments extracted from active compounds (pIC_50_ > 6) and the respective ANN-predicted trypanocidal activity.

Figure 11 shows the structure and biological activity of 35 fragments that were predicted to be the most promising according to their trypanocidal profile. This group is characterized by a diversity of chemical motifs having two to four rings with the predominant groups being pyridine, pyrimidine, benzene, piperazine, triazole, benzothiazole, benzofuran, oxadiazole, and pyrazolopyrimidine. The four most active fragments from this collection have a phenylsulfonyl-piperazine (fragments 11 and 12) or a phenylpiperazine-carboxamide moiety (fragments 13 and 14) linked to two aromatic rings that are either pyridine, pyrimidine, or benzene. Replacing one of these aromatic rings with a hydrogen, such as in 16 and 22, led to a reduction of the biological activity. The same effect was observed for 24 and 27, in which one aromatic ring was kept and the benzene was replaced with a hydrogen. The replacement of the pyridine in compound 20 with a pyrimidine in compound 21 led to a subtle lowering of the pIC_50_ value. Another substitution that affected the biological activity was the exchange between the benzofuran, benzothiazole, and pyrazolopyrimidine in fragments 17, 18, and 19. Among these three compounds, the benzofuran derivative was the most potent. Furthermore, replacing the pyrazolopyrimidine in compound 23 with a benzothiazole in compound 15 increased the trypanocidal activity.

**Figure 12:**
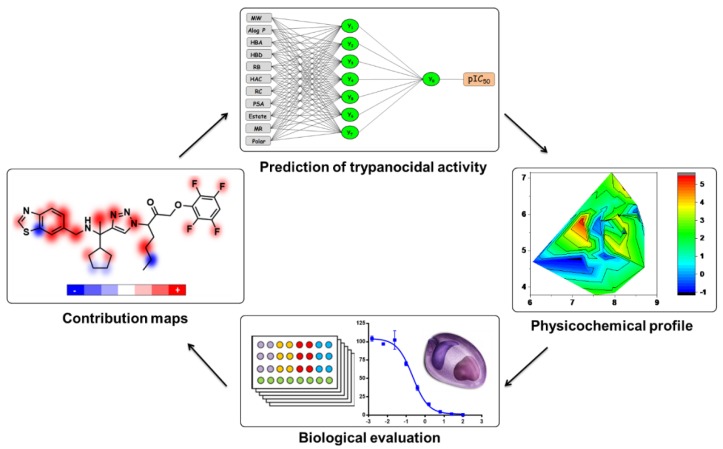
Proposed workflow for the design of novel anti-*Trypanosoma cruzi* compounds based on the generation of contribution maps, prediction of trypanocidal activity using artificial neural networks, and analysis of physicochemical properties. Compounds featuring favorable properties can be synthesized and evaluated against the parasite.

After analyzing the other molecular scaffolds, it is worth mentioning that for fragments 37 and 38, it was not possible to establish a direct relationship between the presence of the oxadiazole group and trypanocidal activity. Replacing the oxadiazole in compound 37 with a phenyl in 44 decreased the pIC_50_ value; however, the same modification involving fragments 35 and 38 produced the opposite effect. Among cyclopentane derivatives 30, 32, and 43, replacing the benzothiazole in fragment 30 with pyrazolopyrimidine and benzofuran in 32 and 43, respectively, decreased the biological activity; the most significant effect occurred for the benzothiazole-benzofuran exchange, which resulted in a decrease of 0.47 in the pIC_50_ value. Finally, the insertion of a methyl cyclopentane moiety at the triazole ring of 41 resulted in fragment 36 and increased the trypanocidal activity. Figure 12 shows the overall scheme for the design of novel trypanocidal compounds based on the workflow proposed in this work.

## 4. Discussion

The physicochemical characterization of the dataset revealed that most compounds follow the Lipinski’s Rule of Five, as illustrated in Figure 3 [65]. The determinant role played by these properties was shown by the analysis of the weights that were attributed to each physicochemical descriptor at the hidden-layer neurons of the best ANN (Table 4). MW, *a*LogP, HBA, and HBD exhibited the greatest difference between the number of positively and negatively modulated neurons. MW positively weighted six (85.7%) out of the seven hidden-layer neurons, and *a*LogP, HBA, and HBD positively weighted five (71.4%) neurons. These four descriptors are closely related to bioavailability and the ability to permeate cell membranes, and therefore, the capacity of a compound to reach its molecular target. The number of rotatable bonds, which had a mean value of 5.93 for the whole dataset, also modulated most neurons in the same way—71.4% of the hidden-layer neurons were negatively weighted by this property. HAC and RC positively weighted four out of seven hidden-layer neurons. The predominantly positive weighting profile of HAC and RC can be associated with that of MW and *a*LogP; an increase in the first two properties generally leads to an increase in the latter two. Another finding worth mentioning is that the KPLS models led to the identification of a set of fragments that are strongly associated with enhanced trypanocidal activity. Most of these fragments contain between two and three rings, which follows the physicochemical profile identified by the ANN and shown in the heat maps for these chemotypes (Figure 9). Aromatic nitrogen-containing rings and fused rings are the most common structural features identified within this collection. Cyclopentane and piperazine are the only representants of aliphatic rings. Functionalized short linkers (from one to four atoms) containing amine, amide, sulfone, or ester groups are found between the cyclic groups. Nonfunctionalized linkers are almost exclusively restricted to methylene groups. Another aspect disclosed in this study was that the heat maps for the favorable fragments showed a more restricted physicochemical space compared to the results for the full molecules. For example, the following physicochemical ranges were predicted to be the most adequate for the fragment collection: MW > 260 Da; *a*LogP: 2–3; PSA: 50–80 Å^2^; E-state: 35–63; MR: 65–115; and Polar: 30–53. These findings can be useful guidelines for monitoring the physicochemical profile in Chagas’ disease drug design efforts using fragment-like compounds as starting points.

## 5. Materials and Methods 

### 5.1. Selection and Construction of the Dataset 

The 363 dataset compounds were selected from 39 articles from the Web of Science after eliminating compounds lacking IC_50_ values, and duplicated, inorganic, and metal-containing molecules [26,27,28,29,30,31,32,33,34,35,36,37,38,39,40,41,42,43,44,45,46,47,48,49,50,51,52,53,54,55,56,57,58,59,60,61,62,63,64]. All structures were built using the default settings of Canvas 2.9 (Schrodinger LLC, New York, NY, USA) [66]. IC_50_ values were converted to pIC_50_ (−log IC_50_). The 363 dataset compounds are available in the supporting information (Table S1).

### 5.2. Characterization of the Chemical and Biological Space

To characterize the structural and activity landscape of the dataset, we carried out a principal component analysis (PCA) using SYBYL-X 2.0 and UNITY fingerprints as molecular descriptors (Certara, Princeton, NJ, USA) [67,68]. The PCA result was converted into a structure similarity map that provides information on the structure and activity profiles of the dataset. To generate the structure similarity map, two principal components were initially extracted and applied as initial coordinates of the map. Next, Tanimoto distances between the molecular fingerprints were computed to plot all points of the map. Based on the structure similarity map, we selected the training (280 structures) and test (83 structures) sets to run the QSAR analyses. The training and test set molecules are available in the supporting information (Table S1).

### 5.3. Physicochemical Descriptors

The physicochemical properties used as input to the ANNs were calculated using Canvas 2.9 (Schrodinger LLC, New York, NY, USA) [66]. The following descriptors were calculated: MW, *a*LogP, HBD, HBA, RB, PSA, E-state, MR, Polar, HAC, and RC.

### 5.4. Backpropagation Artificial Neural Networks

The ANNs were built using the machine learning environment of WEKA 3.6 (University of Waikato, Hamilton, New Zealand) [69]. The calculated physicochemical descriptors and pIC_50_ values for the training set were used to train the ANNs [22]. The ANNs were built using the multilayer backpropagation perceptron and a logistic activation function [70]. After the weights were initially determined for each descriptor and the pIC_50_ values were predicted, the errors between the experimental and predicted values were used to update the weights for the first intermediate layer [23]. During this procedure, the adjustment of the activation function was performed through the partial derivative of the weights. Variations in the learning rate (0.1–0.4), momentum (0.1–0.4), and number of neurons (1–10) were explored to optimize the ANNs.

### 5.5. Molecular Fingerprints and 2D Contribution Maps

The KPLS models were constructed for the training set using Canvas molecular fingerprints (Schrodinger LLC, New York, NY, USA) as molecular descriptors [66]. Four types of fingerprints were explored: linear, dendritic, radial, and molprint2D [24,25,71]. The best KPLS model, which was obtained with molprint2D descriptors, was used to generate the 2D contribution maps in which red, white, and blue indicate positive, neutral, and negative contributions, respectively.

### 5.6. Heat Maps

Heat maps were constructed to delineate the physicochemical profile of 50 fragments that were highlighted as positive for biological activity. The positive fragments were identified using the 2D contributions maps and extracted from compounds that showed pIC_50_ > 6. The physicochemical properties were calculated using Canvas 2.9 (Schrodinger LLC, New York, NY, USA) [66] and used as inputs for the best ANN, which then predicted the pIC_50_ for the fragment collection. Next, heat maps were built, in which the physicochemical properties that are associated with higher pIC_50_ values could be visually assessed.

## 6. Conclusions

The discovery of novel drugs for Chagas’ disease remains an outstanding challenge. Progress in this area requires the design of prototypes that combine activities against the molecular target and appropriate pharmacokinetics. Achieving a suitable balance among these different and occasionally conflicting properties requires the development of candidates with finely adjusted physicochemical properties. In this study, a set of 363 structurally diverse compounds covering a broad interval of trypanocidal activity was used to build highly predictive QSAR models. The final ANN showed high predictive power for test set compounds (*q*^2^ = 0.81) and identified critical physicochemical properties associated with the biological activity of the dataset. The best KPLS model, which yielded a *q*^2^ value of 0.84, highlighted key fragments strongly correlated with the anti-*T. cruzi* activity of the dataset compounds. The integration of the ANN and KPLS analyses enabled the generation of a privileged fragment collection, for which an optimal physicochemical space was determined. The structural information enclosed in the fragment collection along with the delineated physicochemical landscape are valuable information for guiding the design of novel antichagasic agents with improved properties.

## Figures and Tables

**Table 1:** Performance of the ANNs as a function of the learning rate.

		Training Set					Test Set				
LR	Score	*r*²	MAE	RMSE	RAE	RRSE	*q*²	MAE	RMSE	RAE	RRSE
0.1	0.80	0.79	0.65	0.82	65	68	0.85	0.6	0.75	59	61
0.2	0.76	0.80	0.58	0.76	59	64	0.78	0.66	0.84	65	68
0.3	0.77	0.79	0.60	0.77	60	65	0.78	0.67	0.89	66	72
0.4	0.75	0.80	0.58	0.77	59	64	0.77	0.69	0.94	68	76

Note: LR = learning rate; *r*^2^ = correlation coefficient for the training set; *q*^2^ = correlation coefficient for the test set (*r*^2^_pred_); RMSE = root mean square error; MAE = mean absolute error; RAE = relative absolute error; RRSE = root relative squared error; score = (1 − |(*r*^2^ − *q*^2^)|) × *q*^2^.

**Table 2:** Performance of the ANNs as a function of the momentum parameter.

		Training Set					Test Set				
MP	Score	*r*²	MAE	RMSE	RAE	RRSE	*q*²	MAE	RMSE	RAE	RRSE
0.1	0.79	0.78	0.64	0.81	64	68	0.84	0.58	0.73	57	59
0.2	0.80	0.79	0.65	0.82	65	68	0.85	0.6	0.75	59	61
0.3	0.80	0.79	0.66	0.83	66	70	0.85	0.62	0.77	61	63
0.4	0.80	0.79	0.67	0.84	67	71	0.85	0.62	0.78	62	63

Note: MP = momentum parameter; *r*^2^ = correlation coefficient for the training set; *q*^2^ = correlation coefficient for the test set (*r*^2^_pred_); RMSE = root mean square error; MAE = mean absolute error; RAE = relative absolute error; RRSE = root relative squared error; score = (1 − |(*r*^2^ − *q*^2^)|) × *q*^2^.

**Table 3:** Performance of the ANNs as a function of the number of neurons in the hidden layer.

		Training Set					Test Set				
NN	Score	*r*²	MAE	RMSE	RAE	RRSE	*q*²	MAE	RMSE	RAE	RRSE
1	-	0.51	0.86	1.03	87	68	-	-	-	-	-
2	-	0.72	0.69	0.85	69	72	-	-	-	-	-
3	-	0.75	0.69	0.86	69	72	-	-	-	-	-
4	-	0.77	0.68	0.84	68	70	-	-	-	-	-
5	0.78	0.78	0.64	0.81	65	68	0.80	0.64	0.82	63	66
6^#^	0.80	0.79	0.65	0.82	65	68	0.85	0.60	0.75	59	61
7	0.81	0.81	0.57	0.73	56	62	0.81	0.59	0.76	58	62
8	0.76	0.79	0.68	0.85	68	71	0.77	0.69	0.90	68	73
9	0.80	0.80	0.65	0.82	65	68	0.82	0.64	0.81	63	66
10	0.77	0.82	0.58	0.75	58	63	0.79	0.62	0.83	61	67

Note: NN = number of neurons in the hidden layer; *r*^2^ = correlation coefficient for the training set; *q*^2^ = correlation coefficient for the test set (*r*^2^_pred_); RMSE = root mean square error; MAE = mean absolute error; RAE = relative absolute error; RRSE = root relative squared error; score = (1 − |(*r*^2^ − *q*^2^)|) × *q*^2^. ^#^Standard NN.

**Table 4:** Weights attributed to each physicochemical descriptor at the individual hidden-layer neurons.

Neuron	MW	*a*LogP	HBA	HBD	RB	HAC	RC	PSA	E-state	MR	Polar
1	0.51	1.94	0.90	3.34	0.73	0.46	−0.27	−0.28	−0.34	0.99	0.59
2	2.77	−3.96	−2.61	−0.11	−0.23	2.98	−2.21	−2.82	1.04	2.39	3.68
3	−1.42	1.79	0.49	2.12	−0.33	−0.20	2.44	1.55	−0.28	−1.07	−0.60
4	0.59	0.06	0.07	1.05	0.14	0.29	0.44	0.31	0.25	0.06	0.76
5	1.39	−4.91	−6.71	−0.28	−0.79	3.33	4.58	1.08	−0.20	4.85	5.49
6	0.55	2.75	0.94	3.22	−2.00	−0.55	0.66	−5.24	−3.30	1.49	−0.76
7	1.60	3.78	5.40	2.38	−2.72	−2.20	−2.52	−0.14	0.92	−1.10	−2.80

Note: MW = molecular weight; *a*LogP = logarithm of the octanol-water partition coefficient; HBA = hydrogen bond acceptors; HBD = hydrogen bond donors; RB = number of rotatable bonds; HAC = heavy atom count; RC = ring count; PSA = polar surface area; E-state = electrotopological state; MR = molar refractivity; Polar = molecular polarizability.

**Table 5:** Model performance as a function of the fingerprint types used as molecular descriptors to build the kernel-based partial least squares (KPLS) models.

Fingerprint	Score	*q* ^2^	*r* ^2^	RMSE	SD	N
Dendritic	0.76	0.82	0.89	0.40	0.53	3
Linear	0.78	0.83	0.89	0.41	0.51	3
Radial	0.80	0.81	0.80	0.54	0.54	2
Molprint2D	0.82	0.84	0.81	0.52	0.50	3

Note: *q*^2^ = correlation coefficient for the test set (*r*^2^_pred_); *r*^2^ = correlation coefficient for the training set; RMSE = root mean square error; SD = standard deviation; N = number of components; score = (1 − |(*r*^2^ − *q*^2^)|) × *q*^2^.

## Supplementary Materials

Supplementary material can be found at https://www.mdpi.com/1422-0067/20/11/2801/s1.

## Abbreviations

ANN	Artificial Neural Network
KPLS	Kernel-based Partial Least Squares
IC_50_	Concentration of compound that inhibits 50% of the growth of *T. cruzi* in phenotypic assays
pIC_50_	−log IC_50_
QSAR	Quantitative Structure-Activity Relationships
WHO	World Health Organization
MW	Molecular Weight
*a*LogP	Octanol-Water Partition Coefficient
HBD	Hydrogen Bond Donors
HBA	Hydrogen Bond Acceptors
RB	Rotatable Bonds
PSA	Polar Surface Area
E-state	Electrotopological State
MR	Molar Refractivity
Polar	Molecular Polarizability
*T. cruzi*	*Trypanosoma cruzi*
RC	Ring Count
HAC	Heavy Atom Count
