# Supplementary material for: Quantitative Structure–Activity Relationships for Structurally Diverse Chemotypes Having Anti-Trypanosoma cruzi Activity
Source: Int J Mol Sci. 2019 Jun 8;20(11):2801. doi: 10.3390/ijms20112801 (PMC6600563; doi:10.3390/ijms20112801)
Supplement: Supplementary file 1 [file ijms-20-02801-s001.pdf]

Supporting Information

Quantitative structure-activity relationships for structurally diverse chemotypes having anti-*Trypanosoma cruzi* activity

Anacleto S. de Souza [1], Leonardo L. G. Ferreira [1], Aldo S. de Oliveira[1,2], and Adriano D. Andricopulo [1,*]

[1] Laboratory of Computational and Medicinal Chemistry, Center for Research and Innovation in Biodiversity and Drug Discovery, Physics Institute of Sao Carlos, University of Sao Paulo, Sao Carlos-SP, Brazil

[2] Department of Exact Sciences and Education, Blumenal Center, Federal University of Santa Catarina, Blumenau, Brazil

* Correspondence: aandrico@ifsc.usp.br; Tel.: + 55 16 3373-9874

Table S1. Dataset used to build the artificial neural networks and the kernel-based partial least squares models.

Molecule	Structure	pIC_{50}	Reference
3	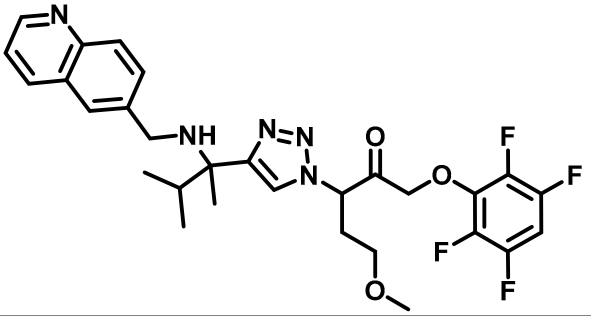	8.70	1
4	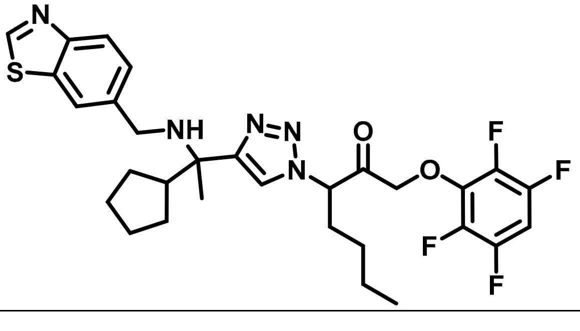	8.52	1

5	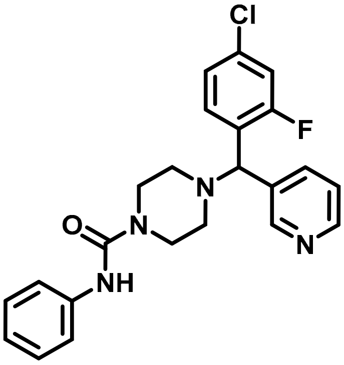	8.22	2
6	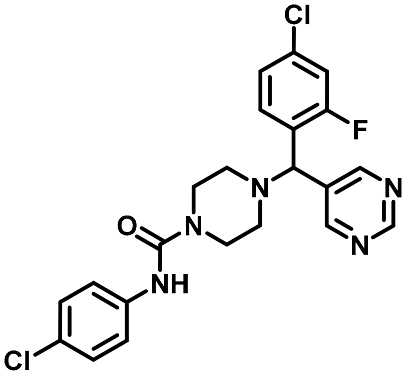	8.00	2
7	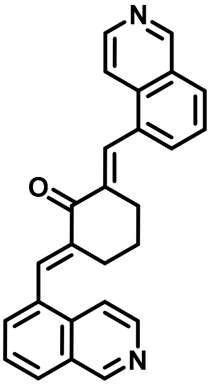	4.16	3
8	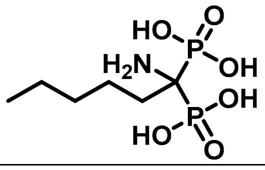	4.11	4
9	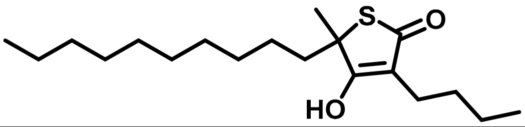	4.11	5
10	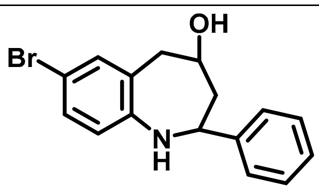	4.01	6
46	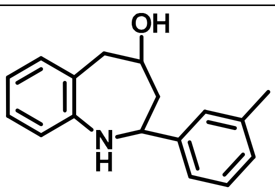	4.01	6

47	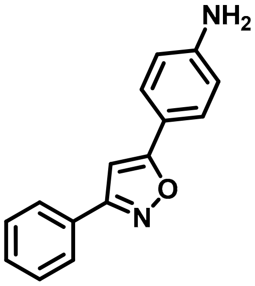	4.04	7
48	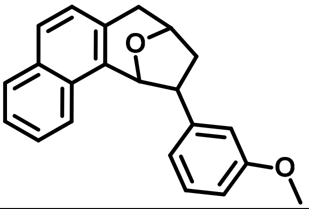	4.05	8
49*	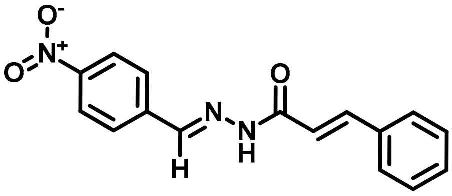	4.09	9
50	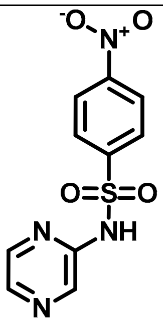	4.12	10
51	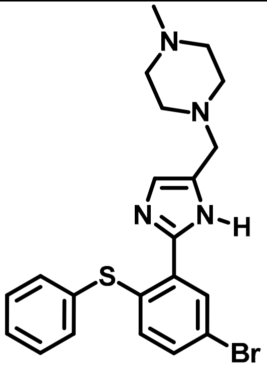	4.12	11
52	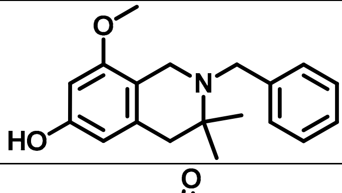	4.12	12
53*	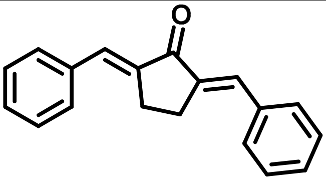	4.14	3

54	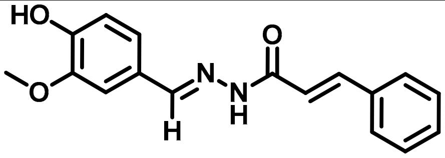	4.14	9
55	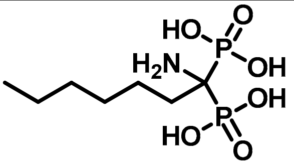	4.14	4
56*	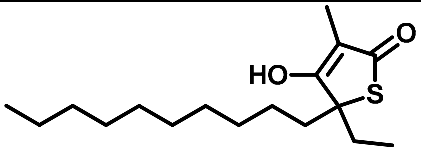	4.14	5
57	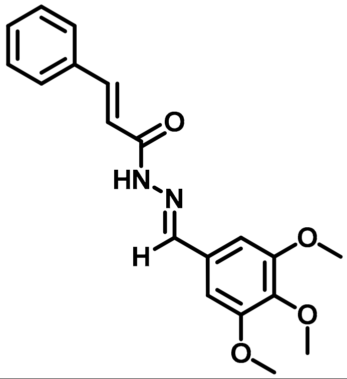	4.15	9
58	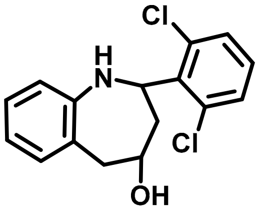	4.16	6
59	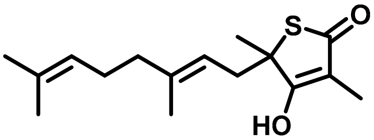	4.17	5
60	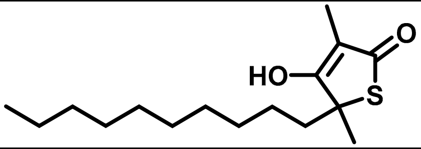	4.17	5
61*	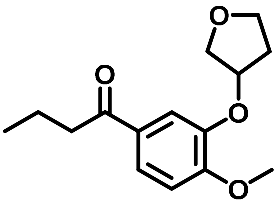	4.17	13
62	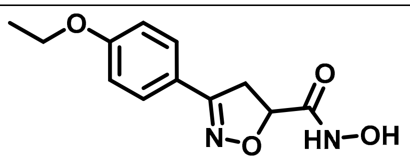	4.18	14

63*	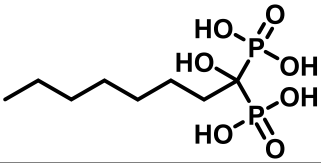	4.18	15
64	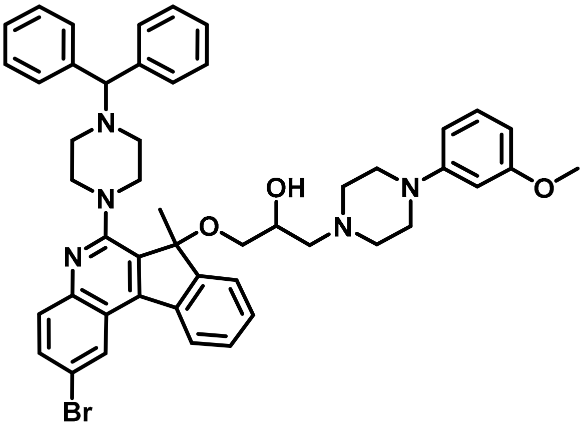	4.19	16
65	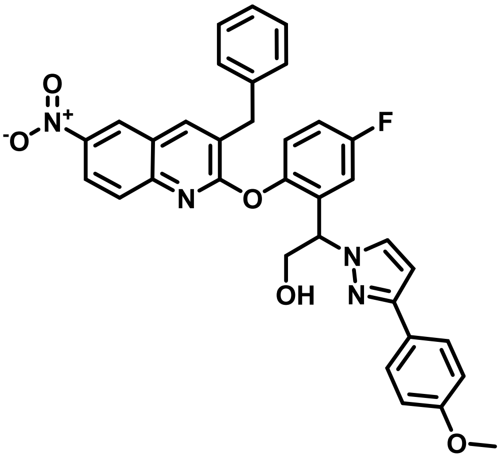	4.19	16
66	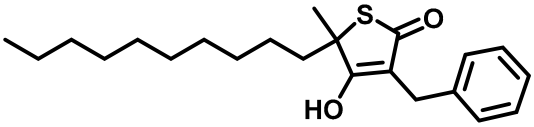	4.19	5
67	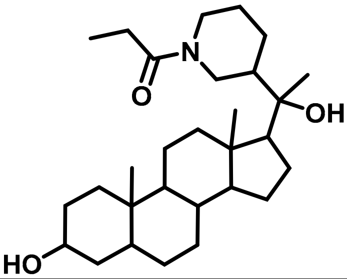	4.19	17
68	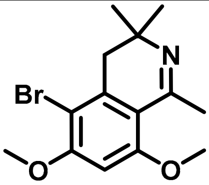	4.20	12
69	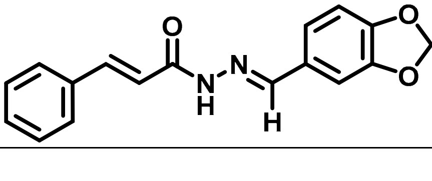	4.21	9

70*	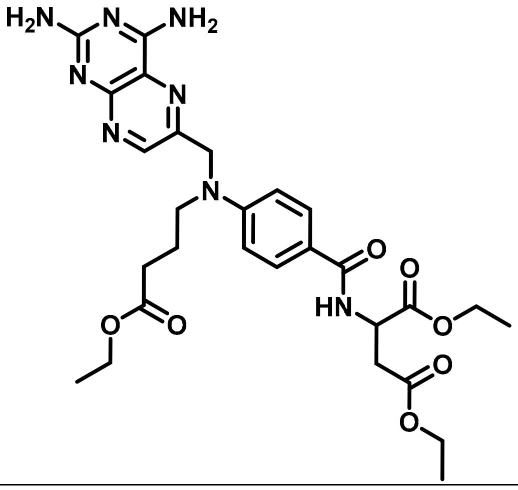	4.21	18
71	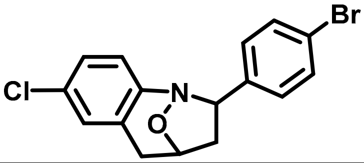	4.22	6
72	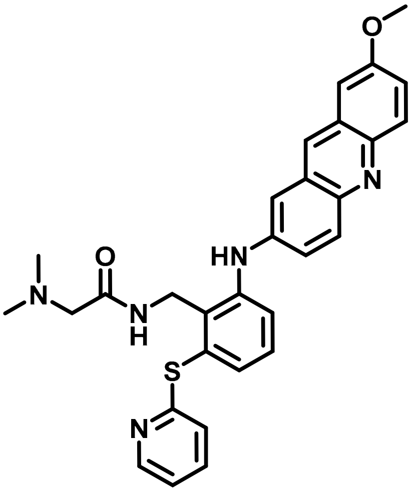	4.24	19
73	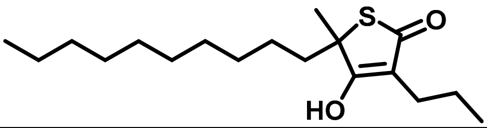	4.25	5
74	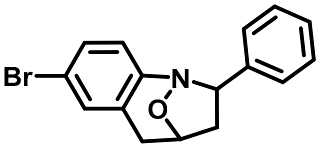	4.25	6
75*	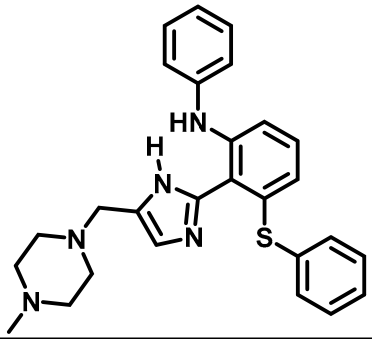	4.27	11

76	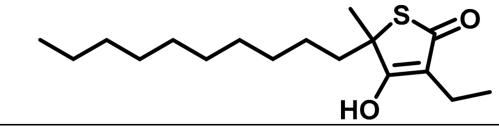	4.27	5
77*	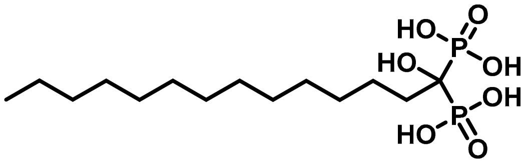	4.27	15
78	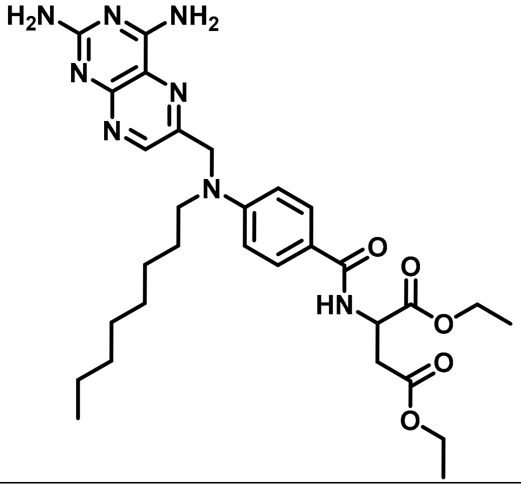	4.27	18
79	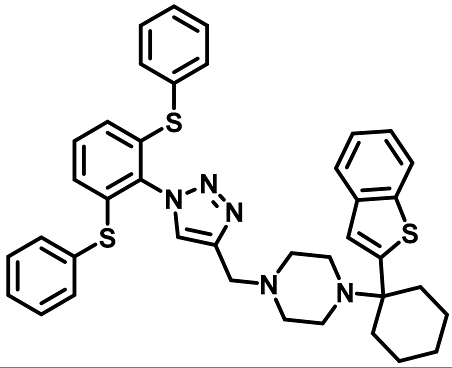	4.28	11
80	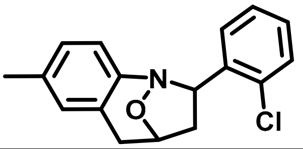	4.28	6
81	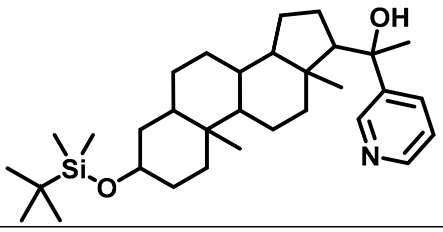	4.29	17
82	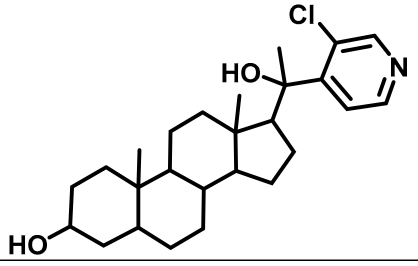	4.30	17

83	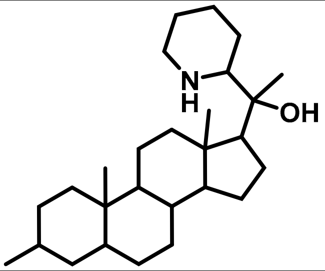	4.30	17
84*	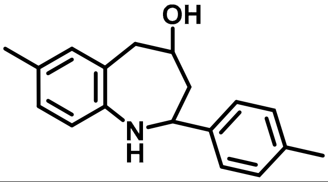	4.30	6
85	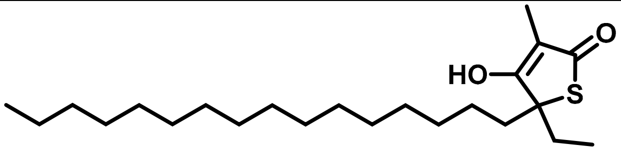	4.31	5
86	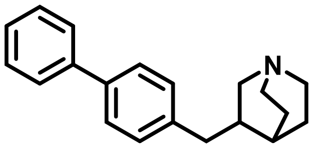	4.33	20
87*	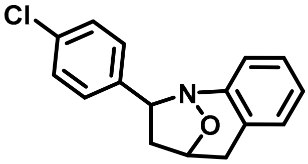	4.34	6
88	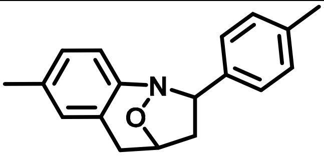	4.34	6
89	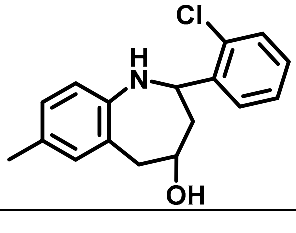	4.34	6
90*	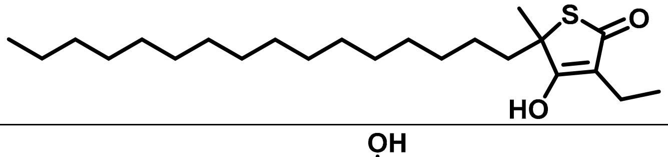	4.37	5
91	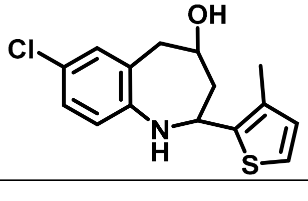	4.38	21

92	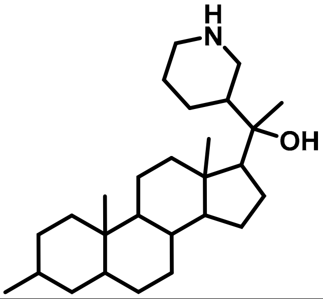	4.38	17
93	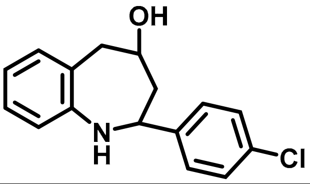	4.38	6
94	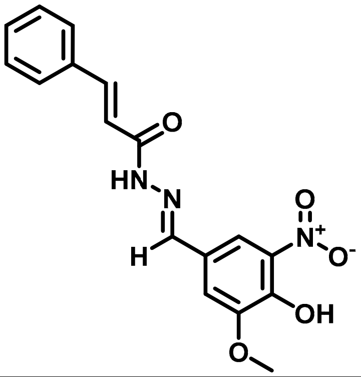	4.40	9
95	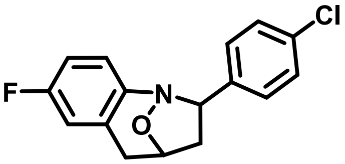	4.40	6
96*	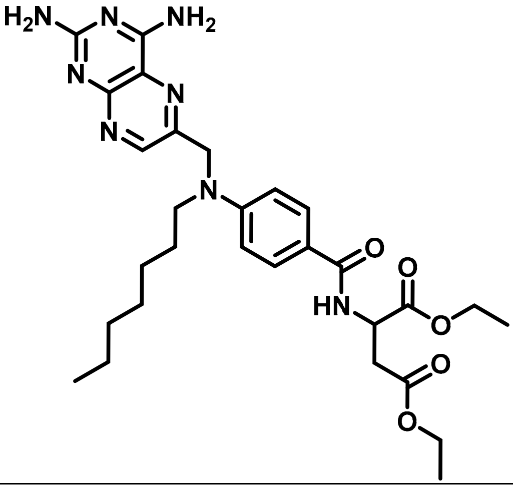	4.41	18
97	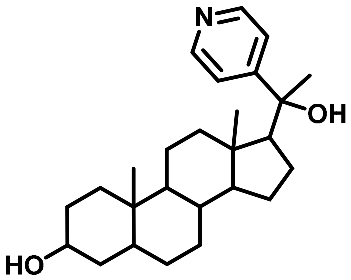	4.46	17

98*	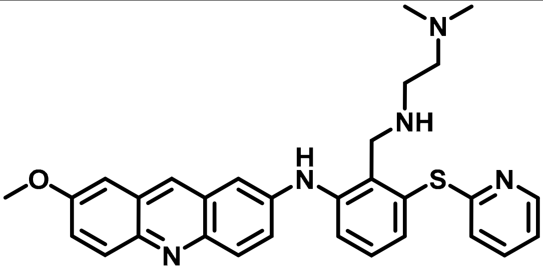	4.47	19
99	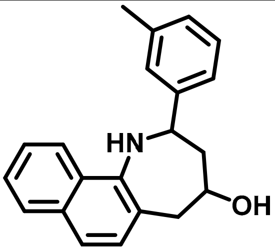	4.48	8
100	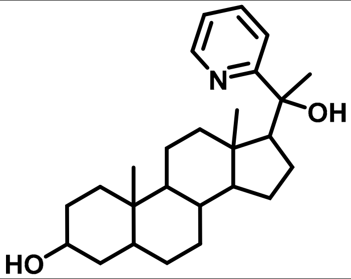	4.48	17
101	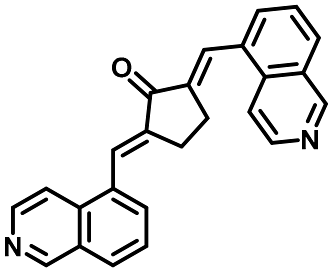	4.50	3
102	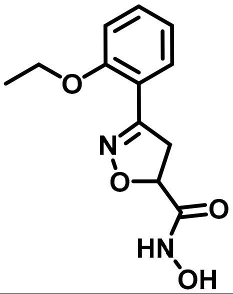	4.52	14
103	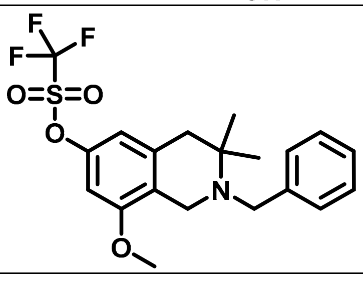	4.55	12

104*	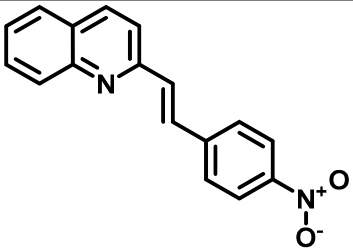	4.55	22
105	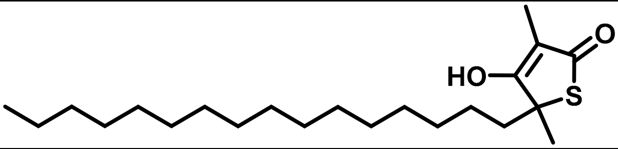	4.55	5
106	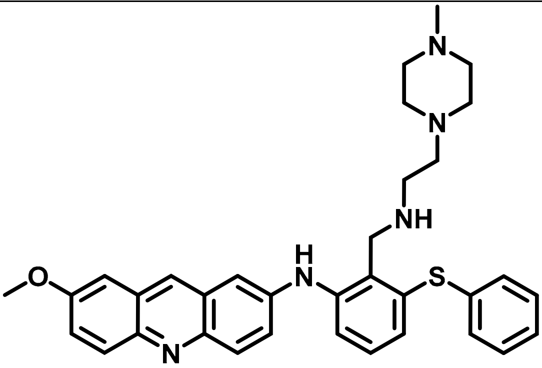	4.56	19
107	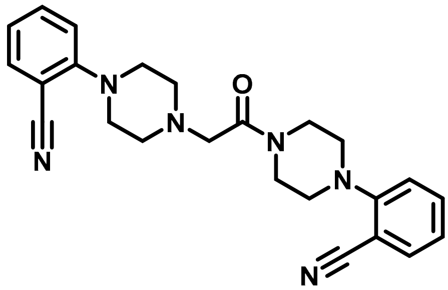	4.59	23
108	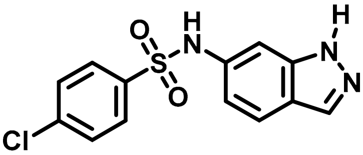	4.59	10
109*	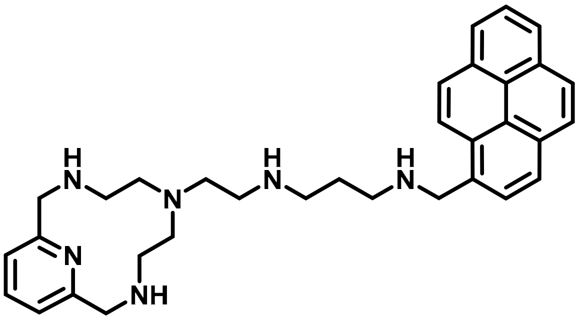	4.60	24
110	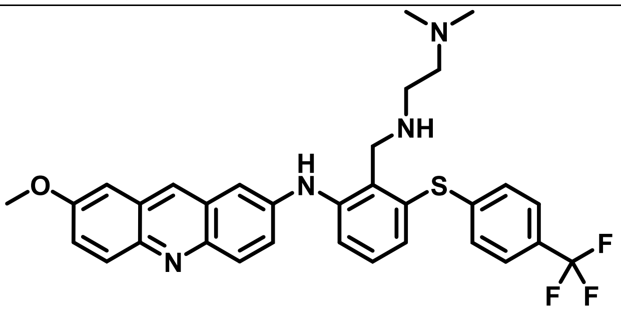	4.61	19

111	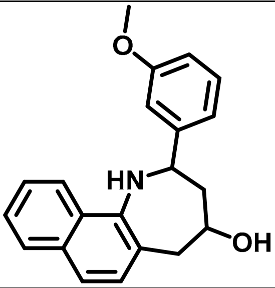	4.61	8
112*	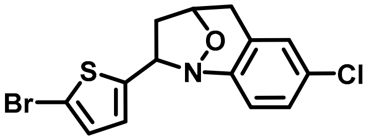	4.62	21
113	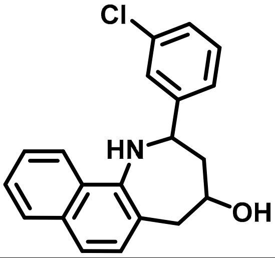	4.63	8
114	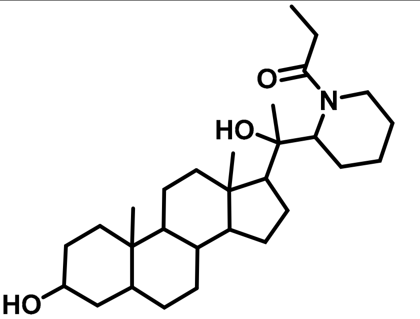	4.64	17
115	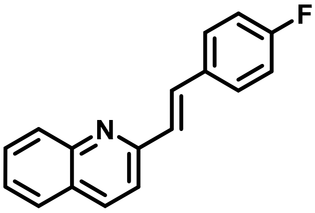	4.66	22
116*	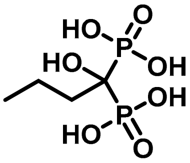	4.67	15
117	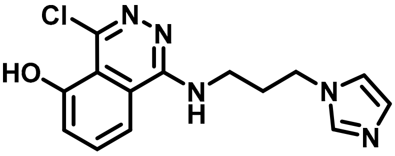	4.68	25

118	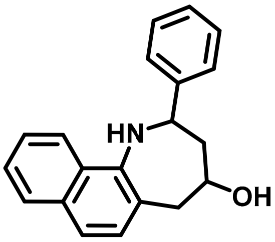	4.71	8
119	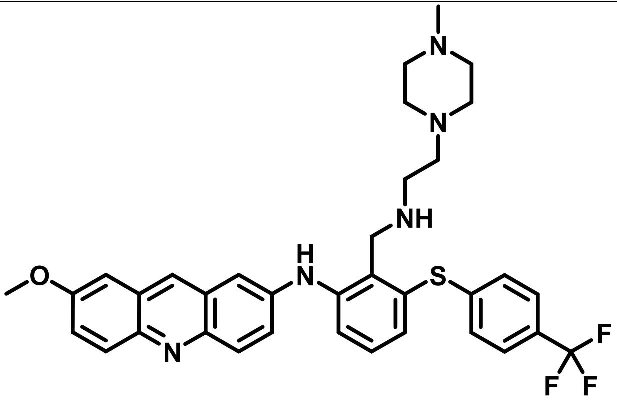	4.72	19
120	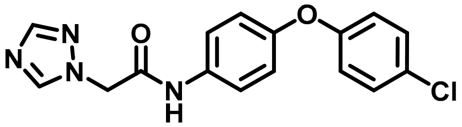	4.74	26
121	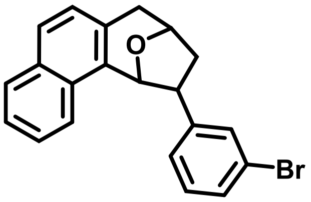	4.74	8
122*	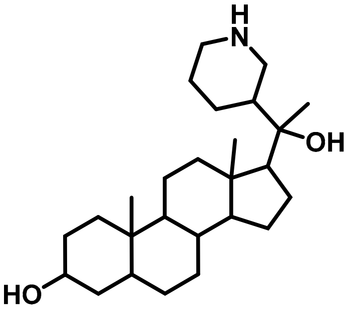	4.74	17
123	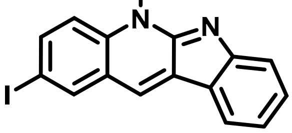	4.74	27
124	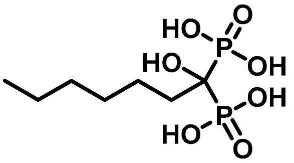	4.74	15

125	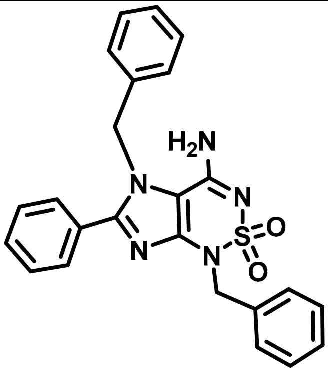	4.77	28
126	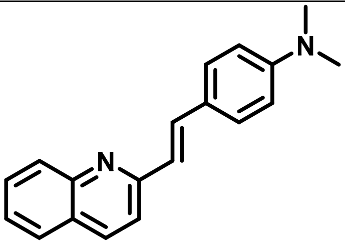	4.77	22
127*	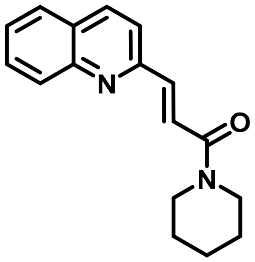	4.77	22
128	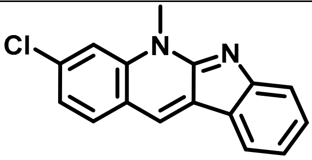	4.77	27
129	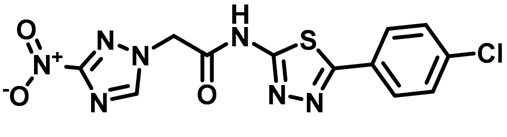	4.78	29
130	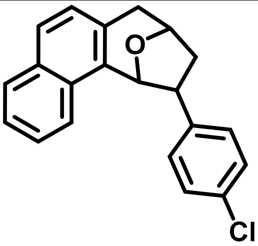	4.79	8
131*	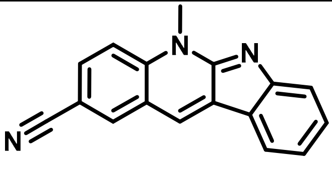	4.79	27

132	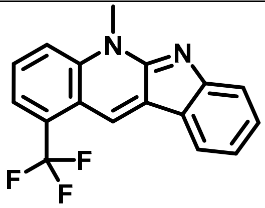	4.79	27
133	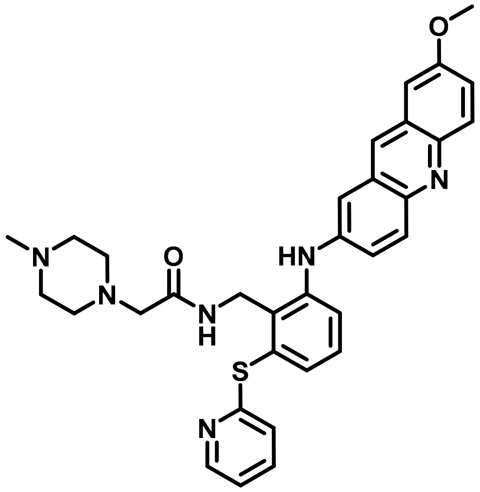	4.80	19
134	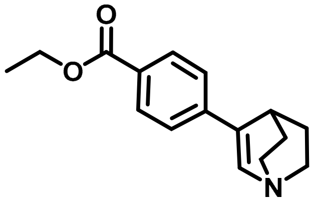	4.80	20
135*	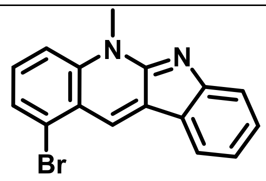	4.80	27
136	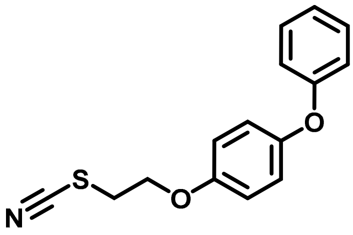	4.80	15
137	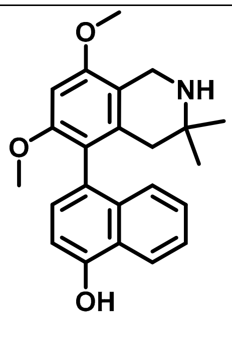	4.82	12

138*	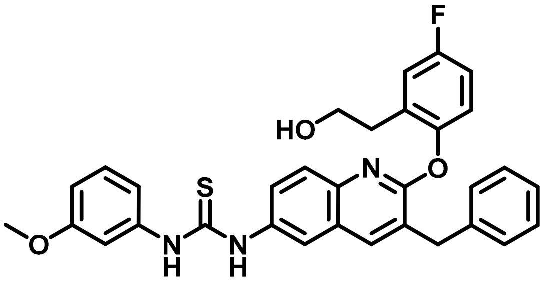	4.83	16
139	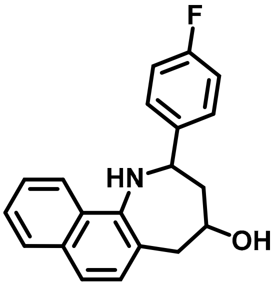	4.83	8
140	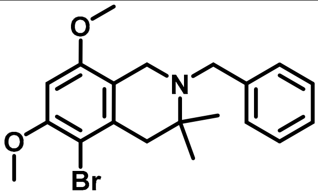	4.83	12
141	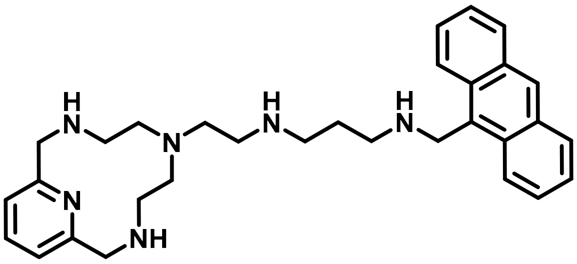	4.85	24
142	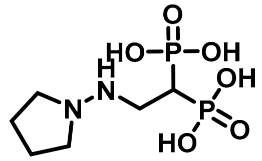	4.85	30
143	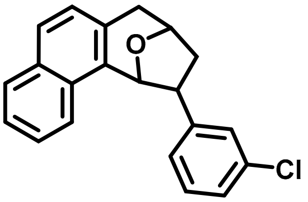	4.88	8

144*	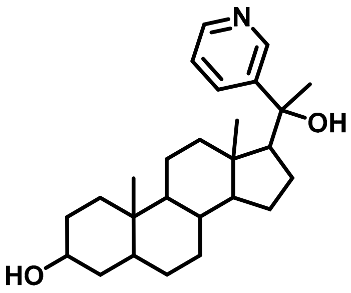	4.88	17
145	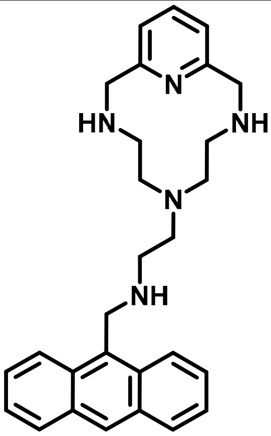	4.89	24
146	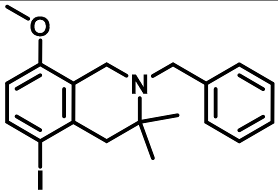	4.89	12
147	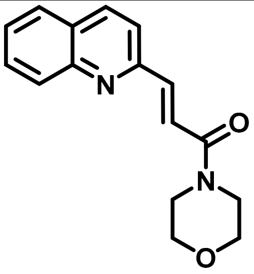	4.89	22
148	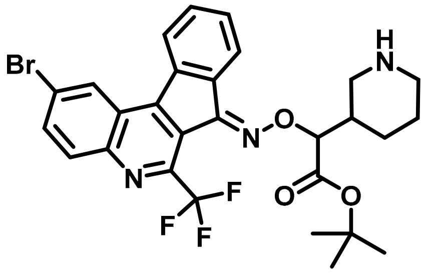	4.91	16

149	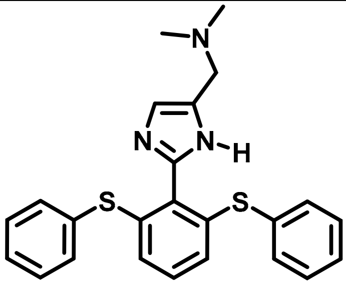	4.91	11
150*	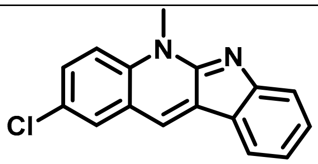	4.92	27
151*	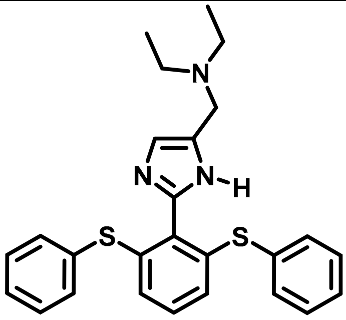	4.94	11
152	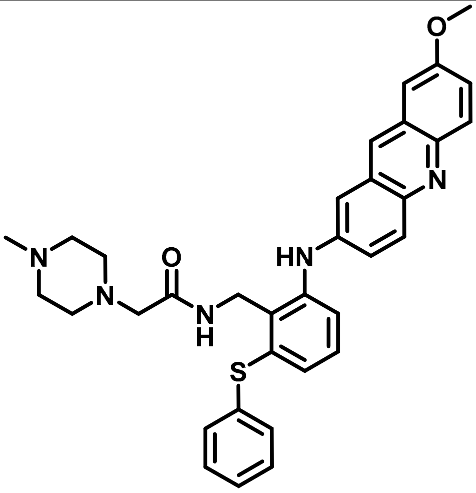	4.95	19
153	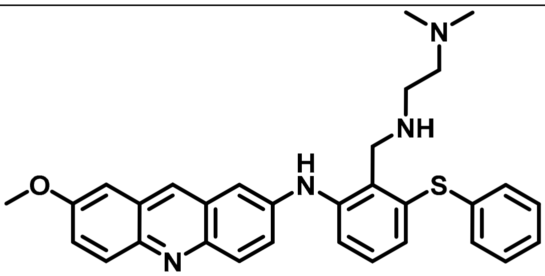	4.95	19

154	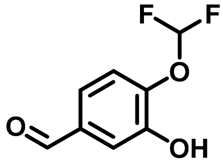	4.96	13
155*	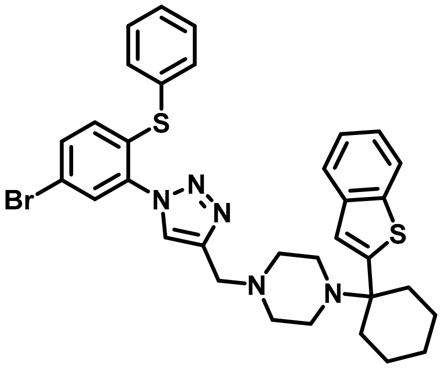	4.97	11
156	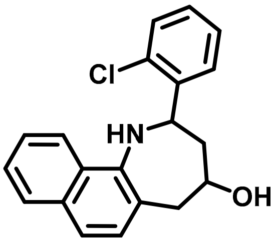	4.97	8
157	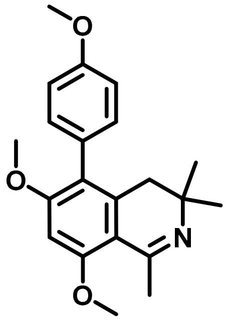	4.97	12
158	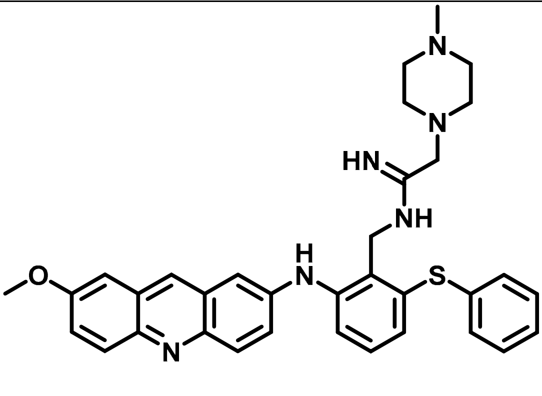	4.99	19

159	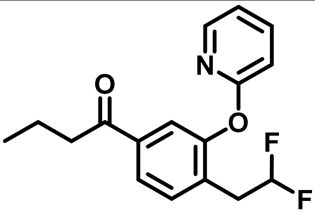	4.99	13
160	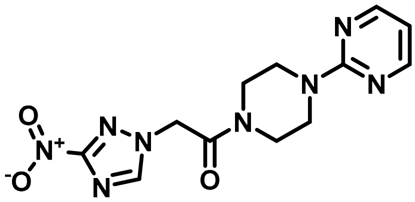	5.00	23
161	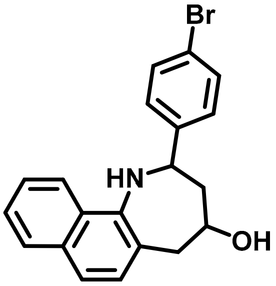	5.00	8
162	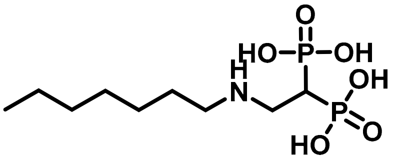	5.00	30
163*	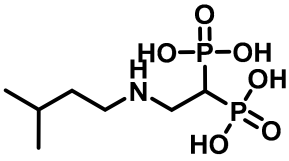	5.00	30
164	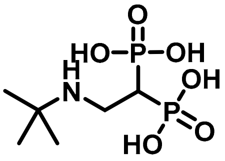	5.00	30
165	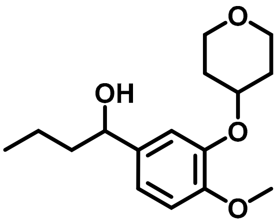	5.00	13

166*	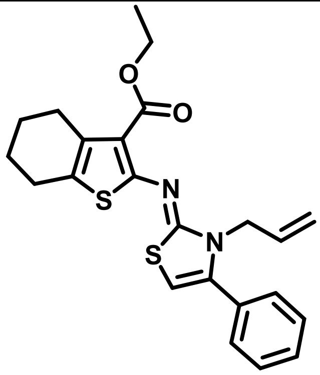	5.01	31
167	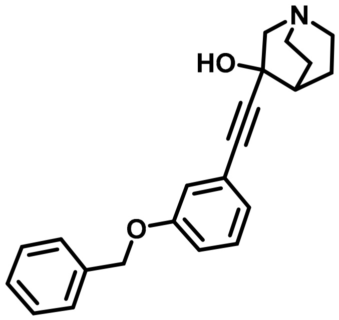	5.02	20
168	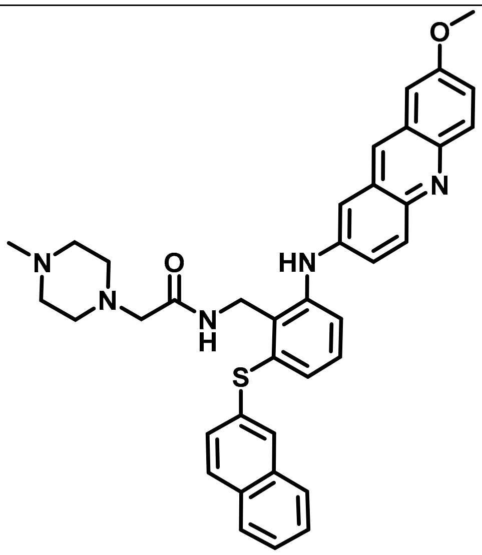	5.03	19

169	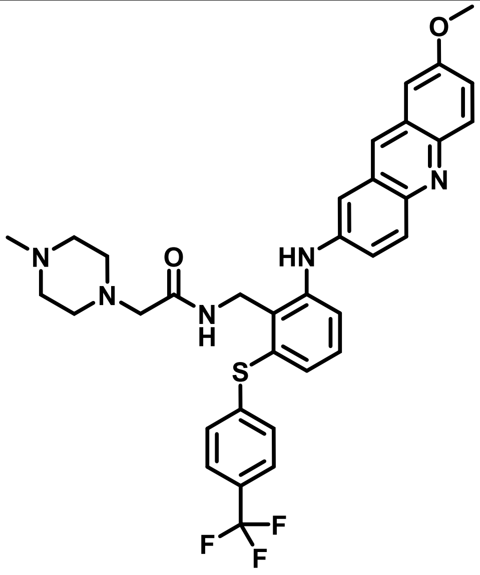	5.03	19
170*	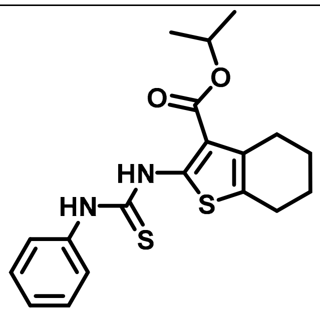	5.06	31
171	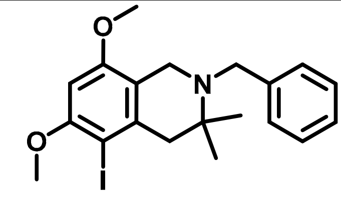	5.07	12
172	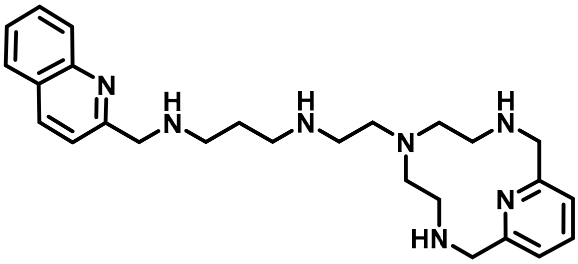	5.08	24
173	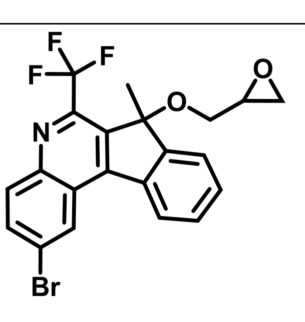	5.08	16

174*	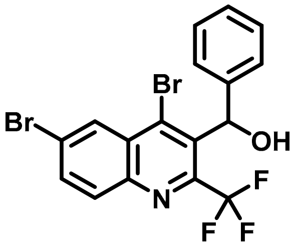	5.09	16
175	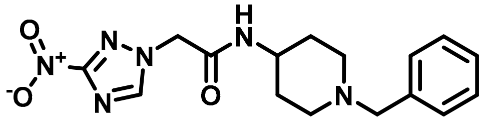	5.10	26
176	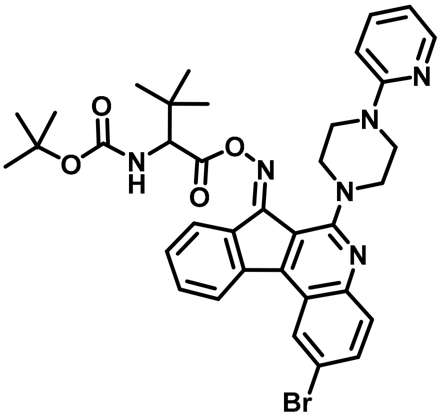	5.10	16
177	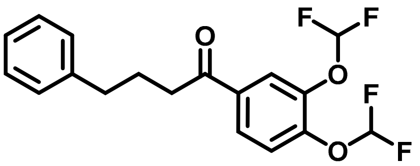	5.11	13
178*	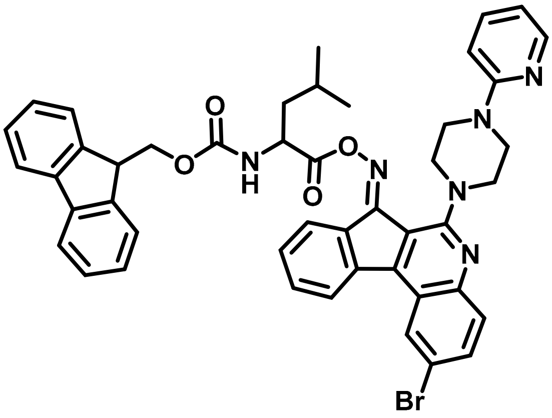	5.12	16
179	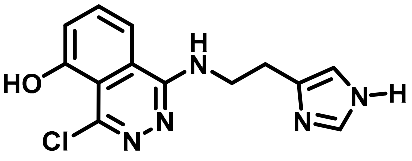	5.13	25

180	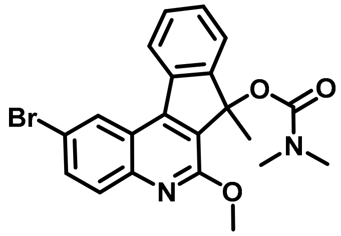	5.13	16
181	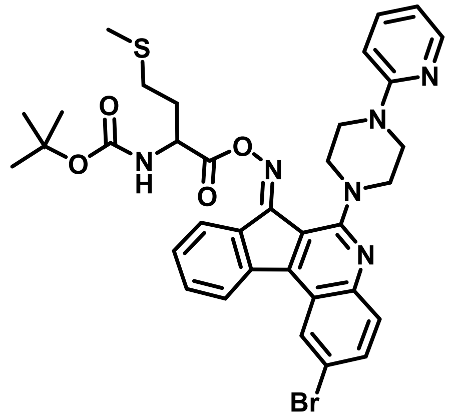	5.13	16
182	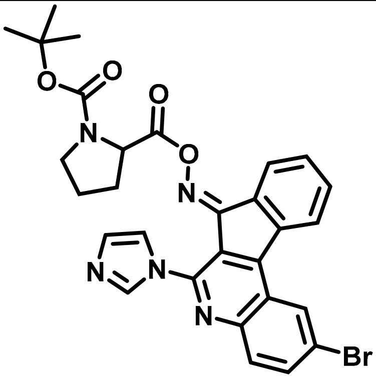	5.13	16
183	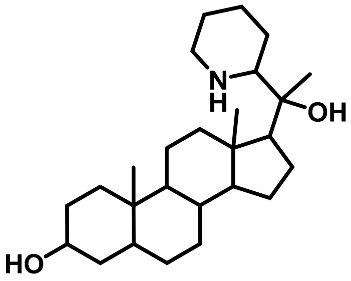	5.13	17
184*	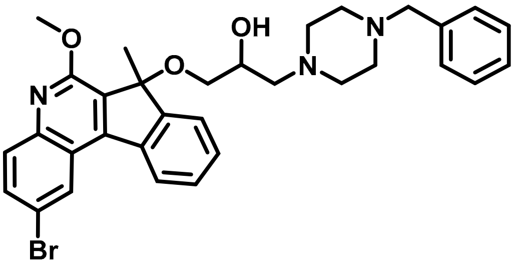	5.14	16

185	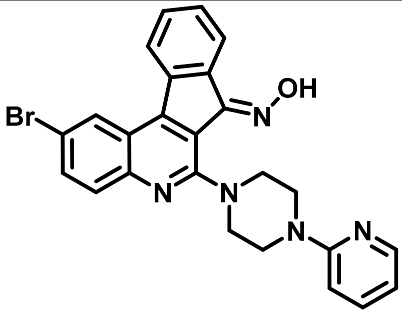	5.15	16
186	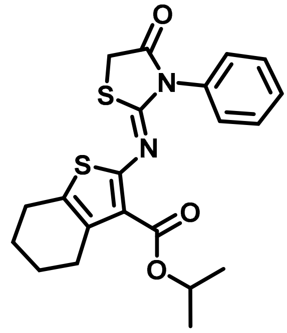	5.16	31
187*	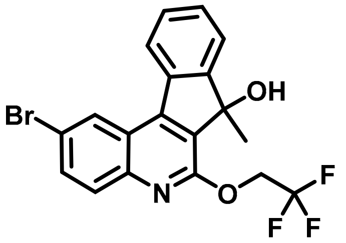	5.16	16
188	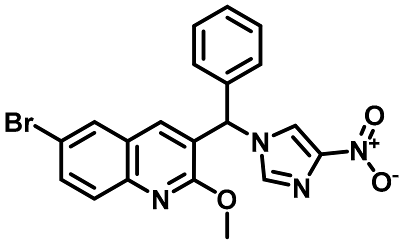	5.16	16
189	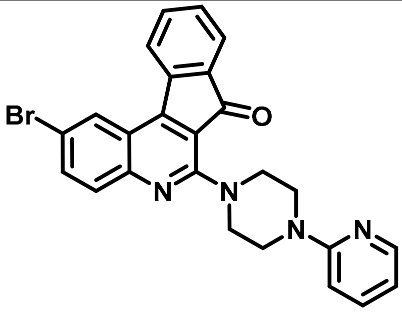	5.16	16

190*	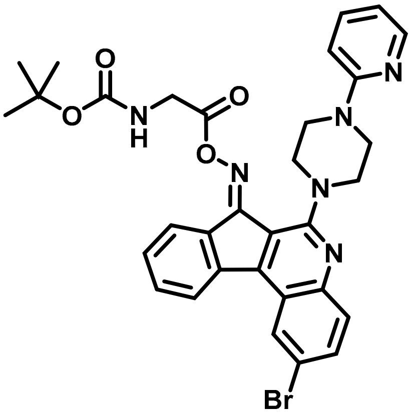	5.17	16
191	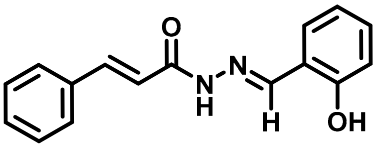	5.18	9
192	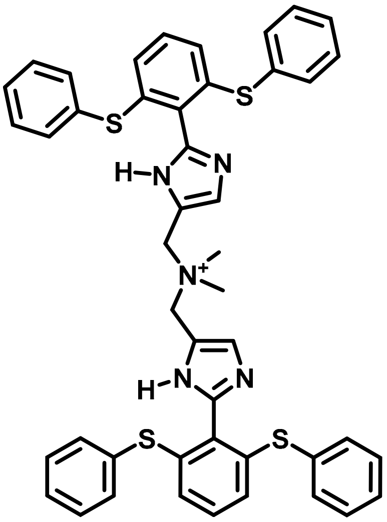	5.20	11
193	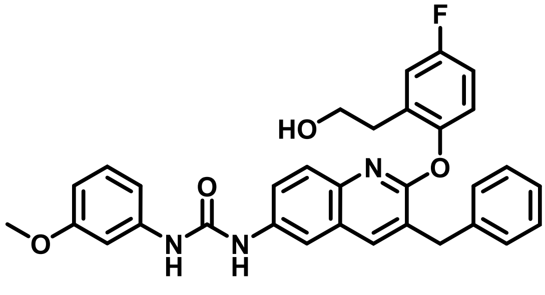	5.21	16

194	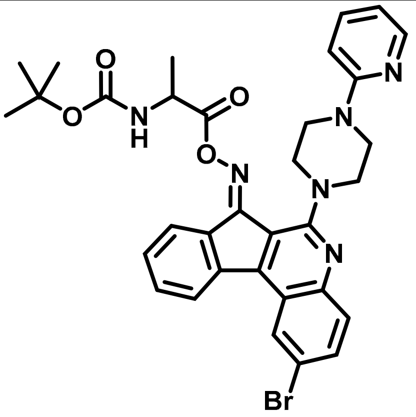	5.21	16
195*	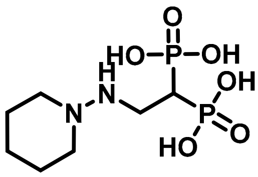	5.21	30
196	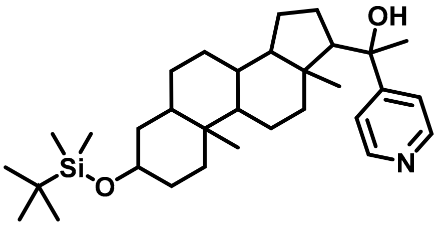	5.21	17
197	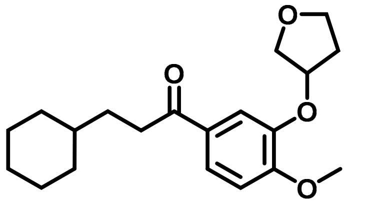	5.21	13
198	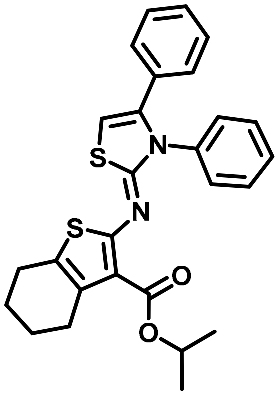	5.22	31
199	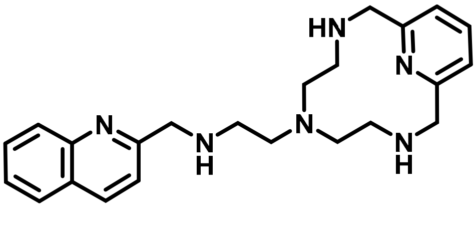	5.22	24

200	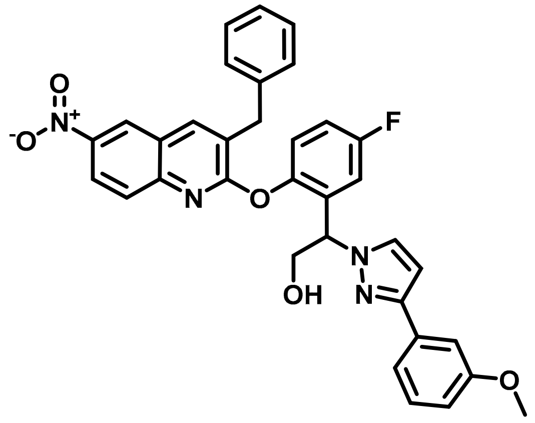	5.22	16
201*	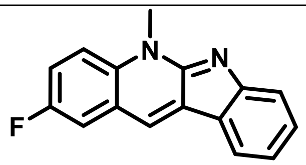	5.22	27
202	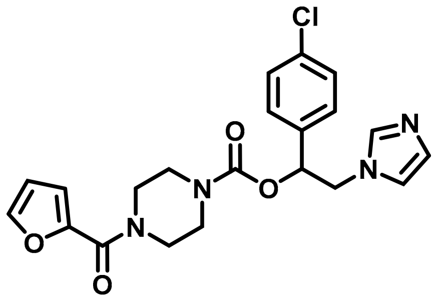	5.23	32
203	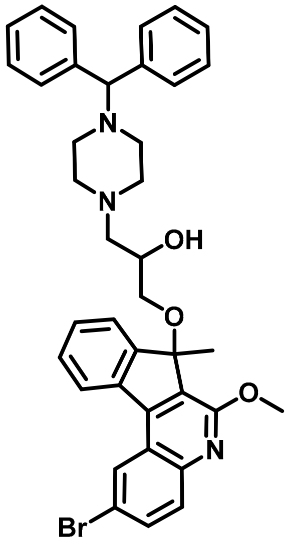	5.23	16
204	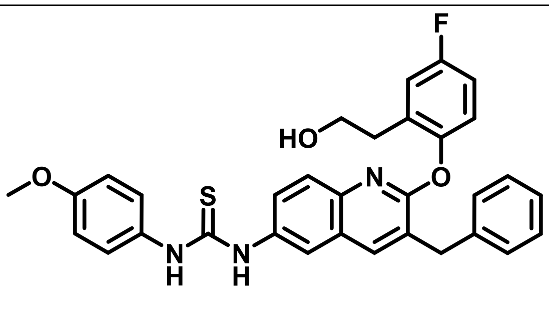	5.24	16

205	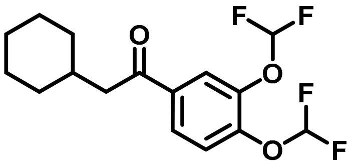	5.24	13
206	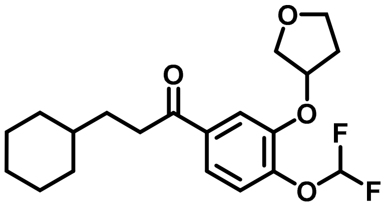	5.24	13
207*	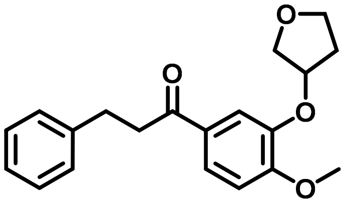	5.25	13
208	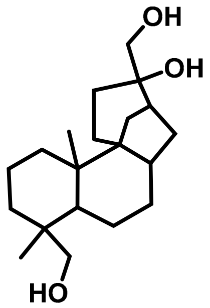	5.26	33
209	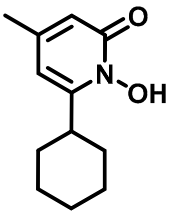	5.27	7
210*	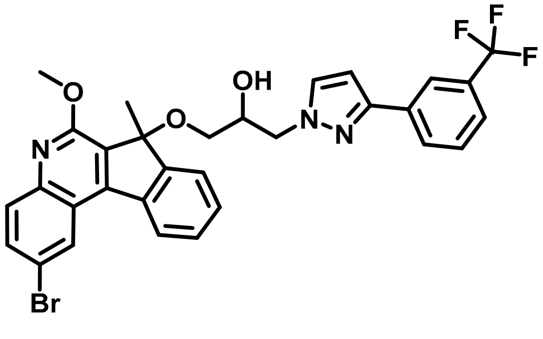	5.27	16

211	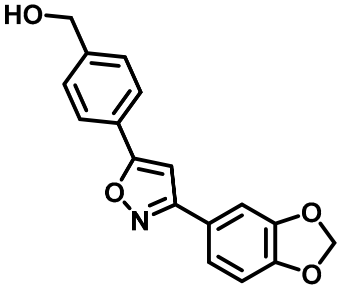	5.28	7
212	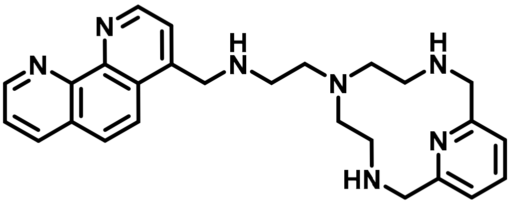	5.28	24
213*	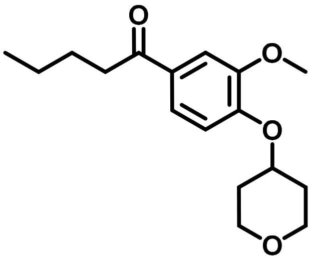	5.28	13
214	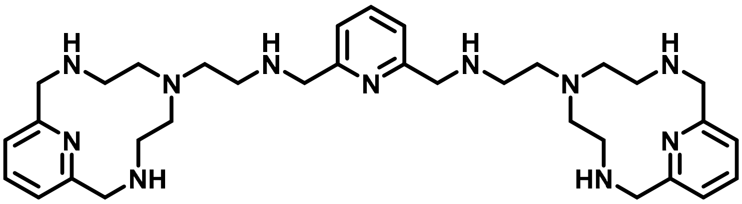	5.29	24
215	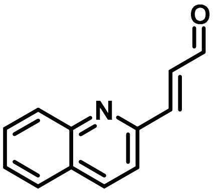	5.30	22
216	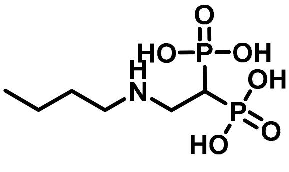	5.32	30
217	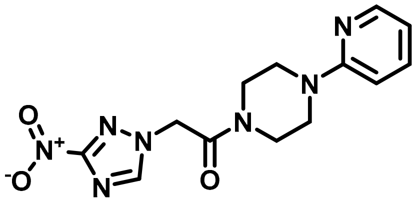	5.33	23

218*	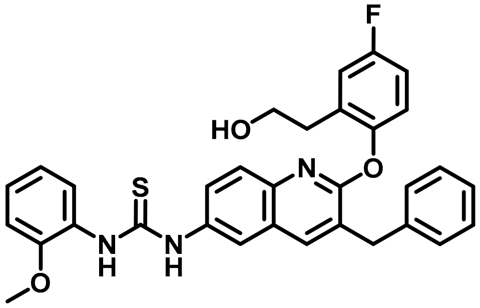	5.36	15
219	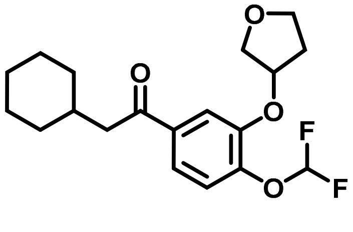	5.36	13
220	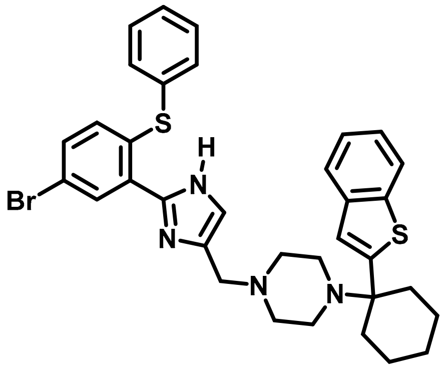	5.37	11
221	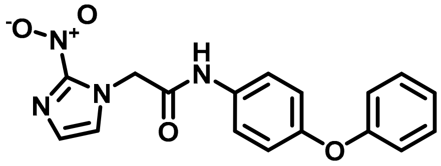	5.38	26
222	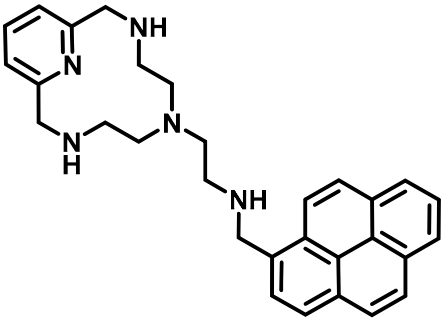	5.38	24
223	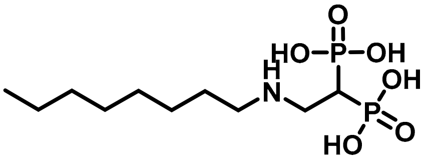	5.39	30

224*	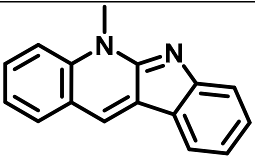	5.40	27
225	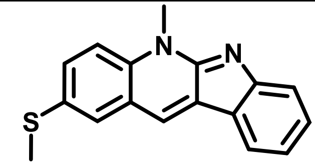	5.40	27
226*	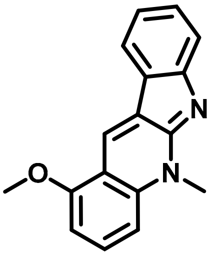	5.40	27
227	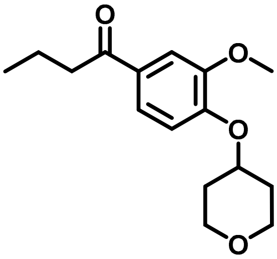	5.40	13
228	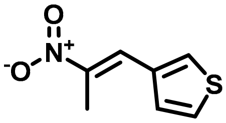	5.41	34
229	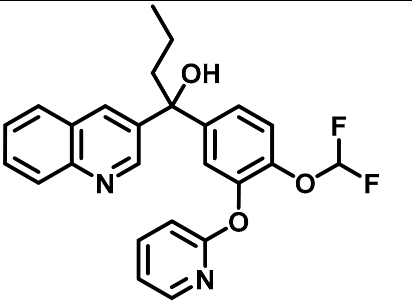	5.42	13
230	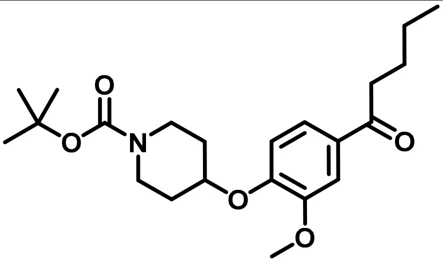	5.42	13

231	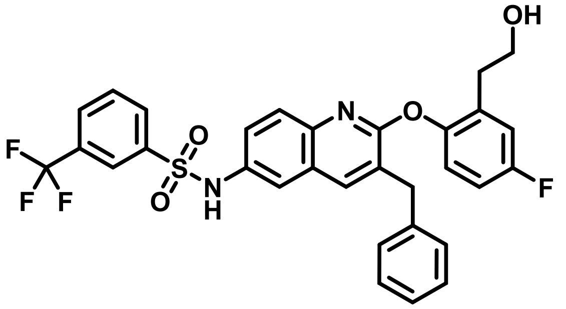	5.43	16
232*	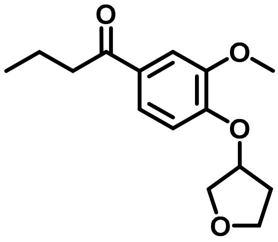	5.43	13
233	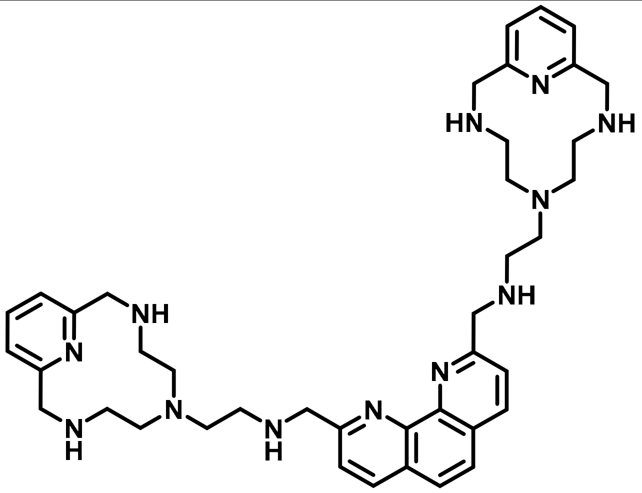	5.44	24
234	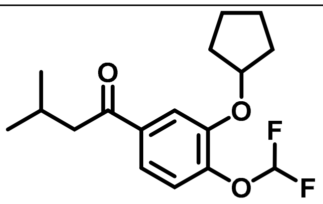	5.44	13
235	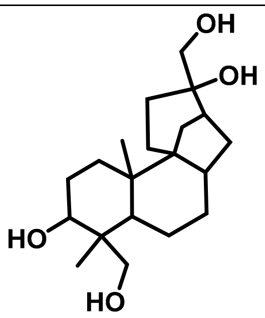	5.46	33

236*	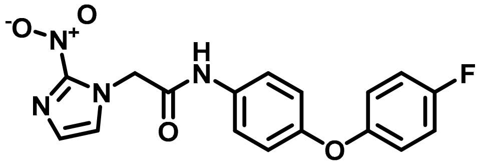	5.48	26
237	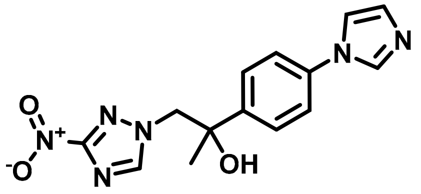	5.48	29
238	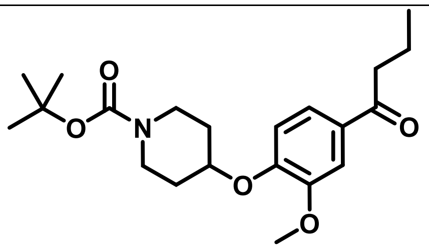	5.48	13
239	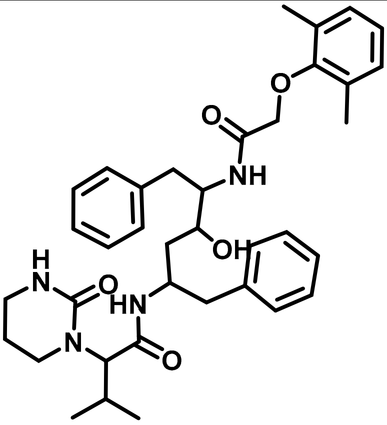	5.49	35
240	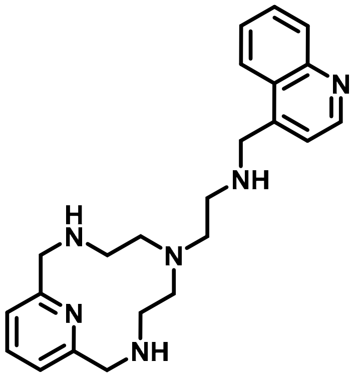	5.49	24

241*	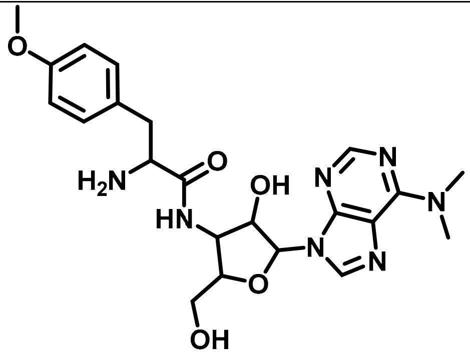	5.50	36
242	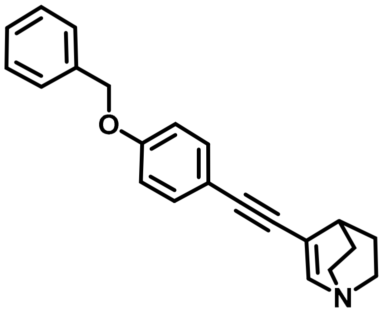	5.51	20
243	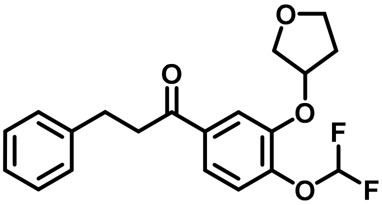	5.51	13
244	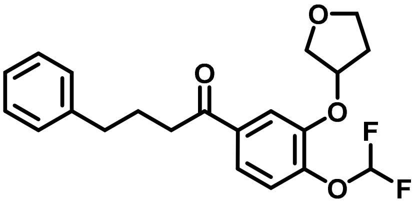	5.51	13
245*	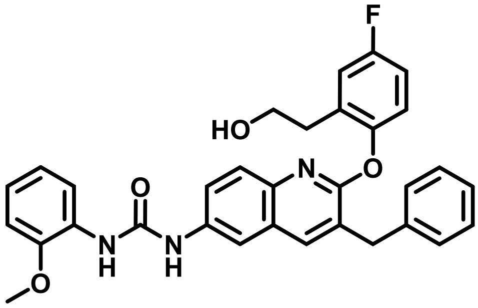	5.54	16
246	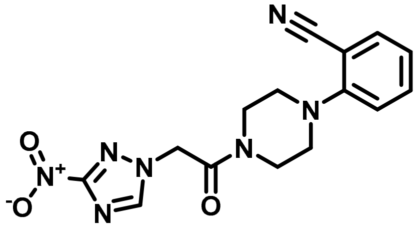	5.55	23

247*	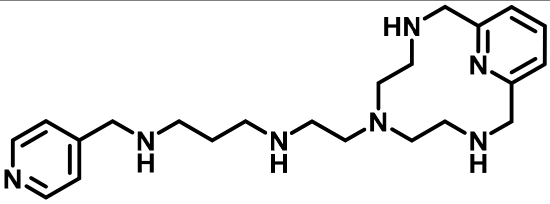	5.55	24
248	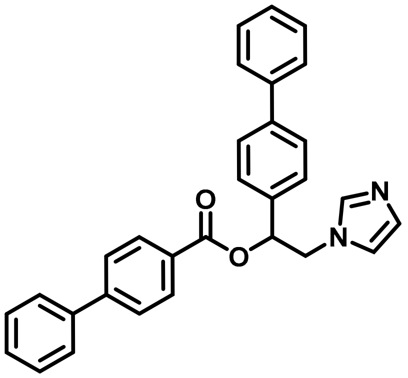	5.57	32
249	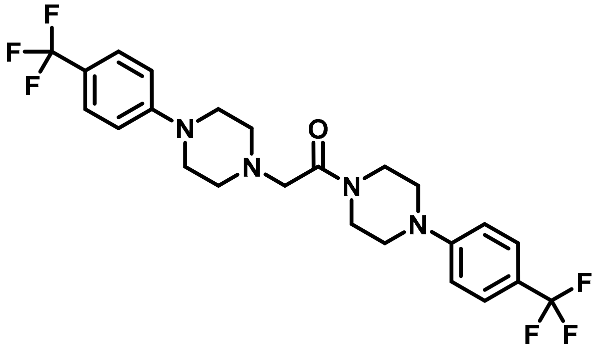	5.57	23
250	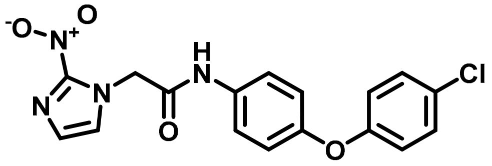	5.59	26
251*	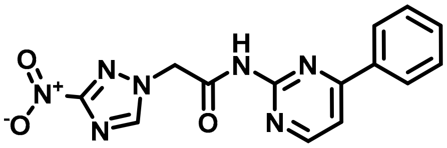	5.60	26
252	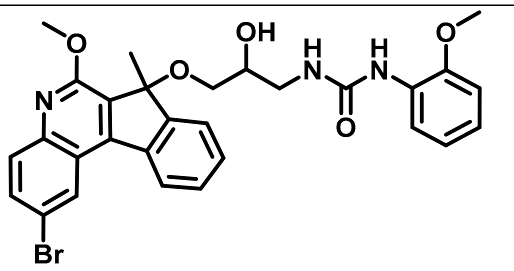	5.60	16

253	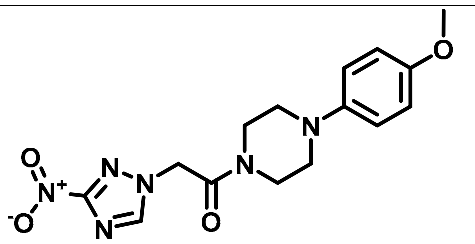	5.62	23
254	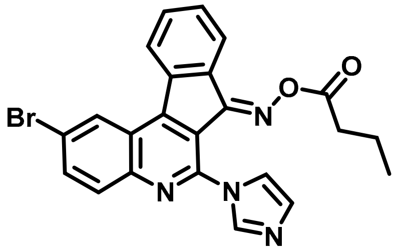	5.62	16
255	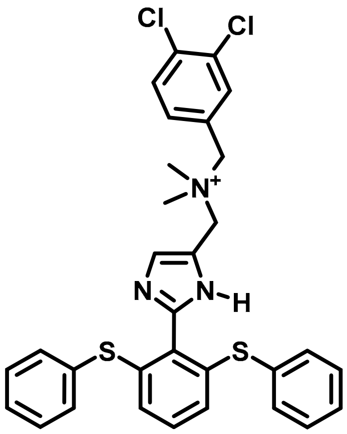	5.62	11
256	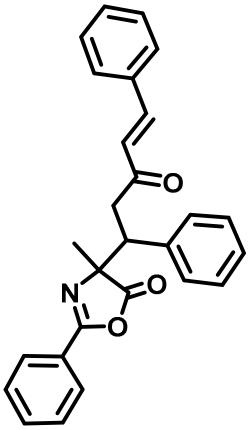	5.63	37
257	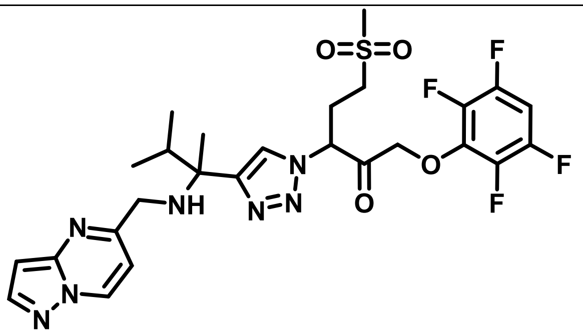	5.63	1

258*	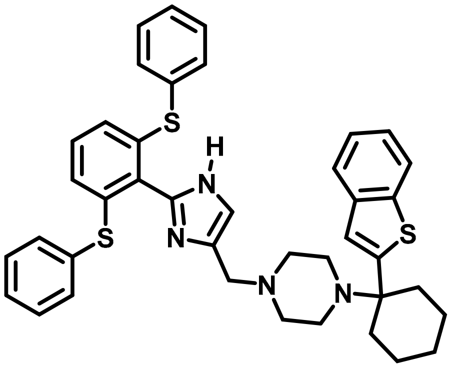	5.63	11
259	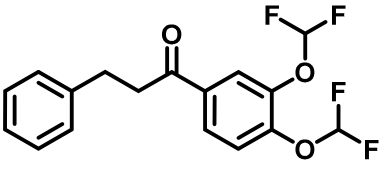	5.64	13
260	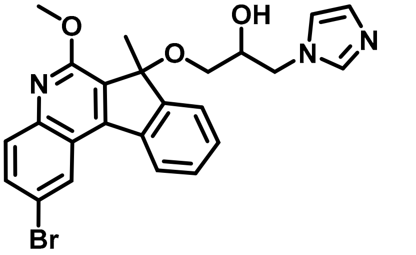	5.67	16
261	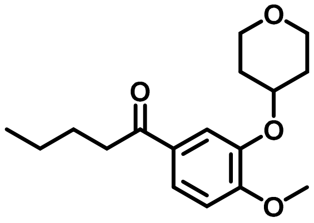	5.68	13
262*	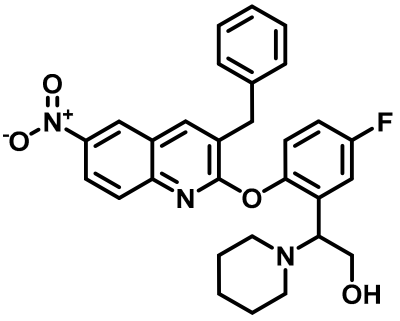	5.70	16

263*	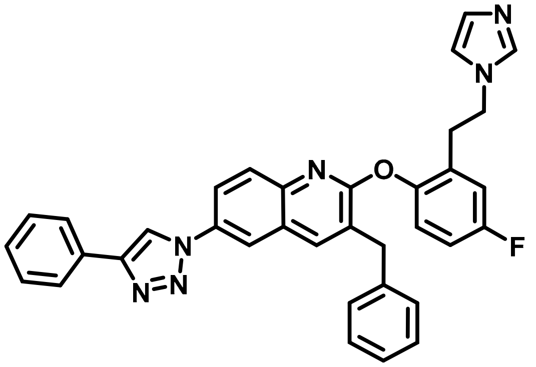	5.71	16
264	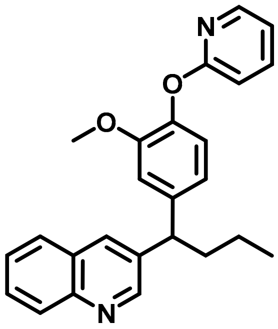	5.72	13
265	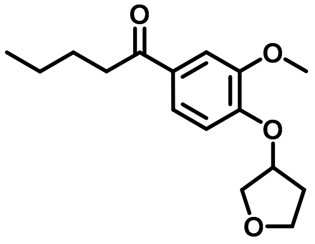	5.74	13
266*	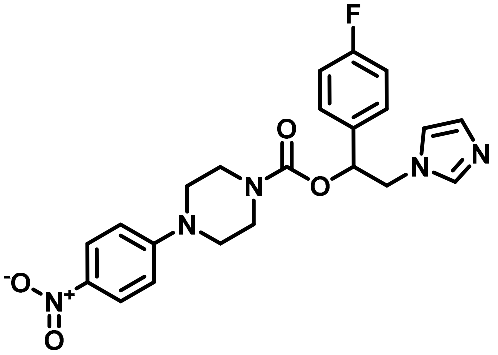	5.77	32
267	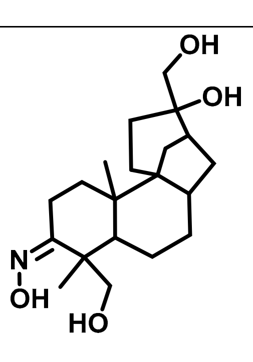	5.77	33

268	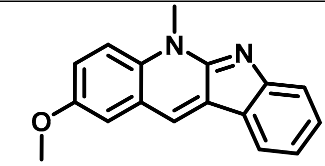	5.77	27
269*	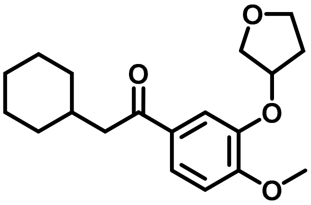	5.77	13
270	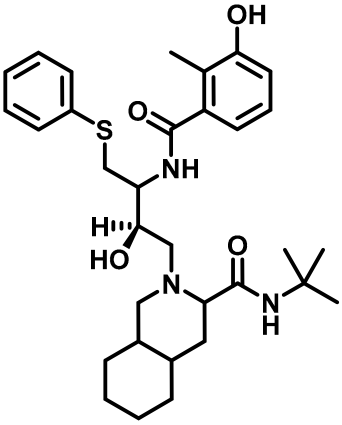	5.80	35
271	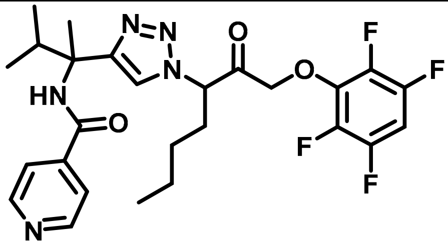	5.80	1
272	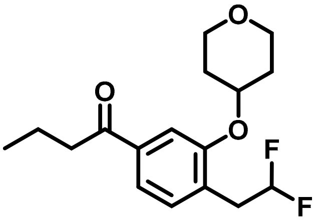	5.80	13
273*	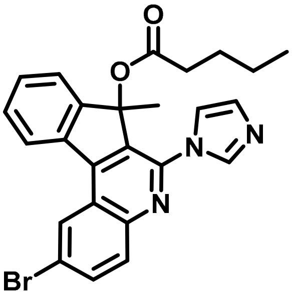	5.84	16

274	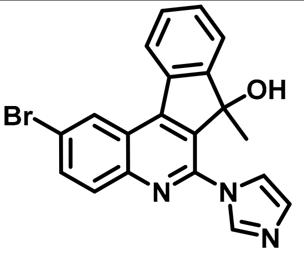	5.86	16
275	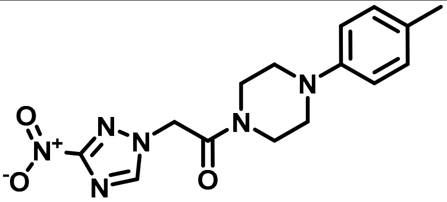	5.88	23
276	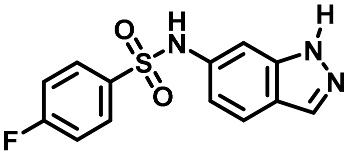	5.89	10
277	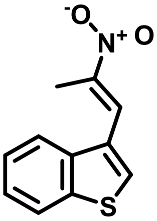	5.89	34
278	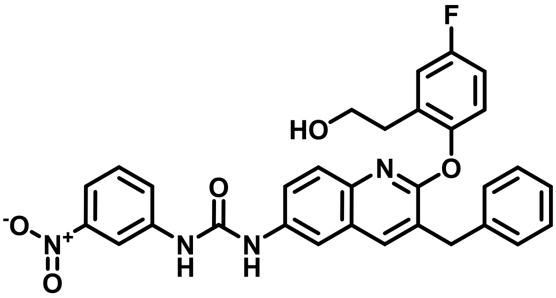	6.00	16
279	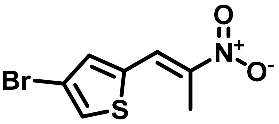	6.00	34
280	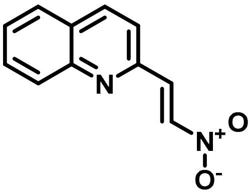	6.00	22

281*	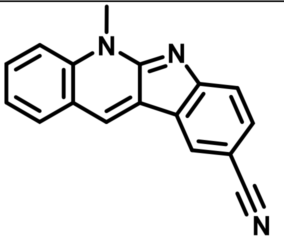	6.00	27
282	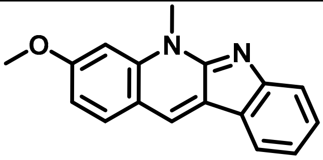	6.00	27
283*	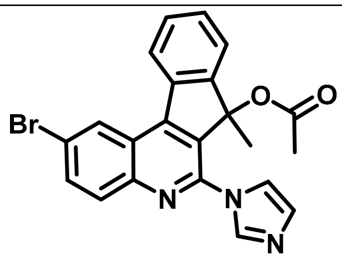	6.03	16
284	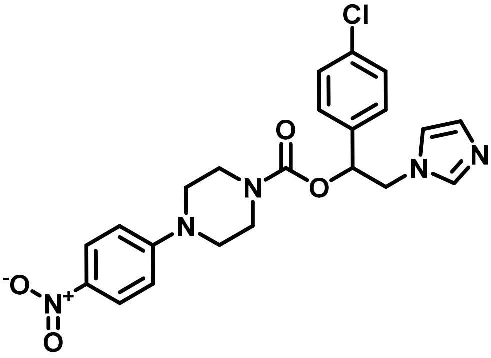	6.06	32
285**	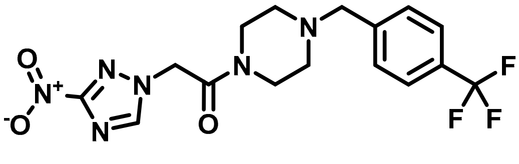	6.06	23
286	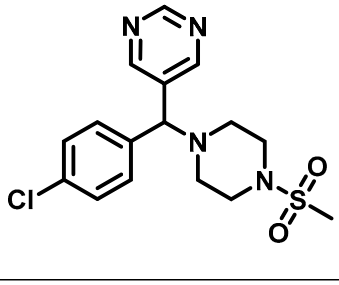	6.06	2
287	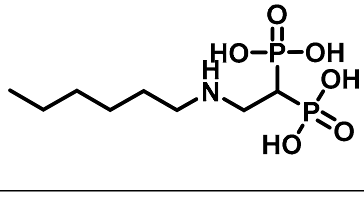	6.08	30

288*	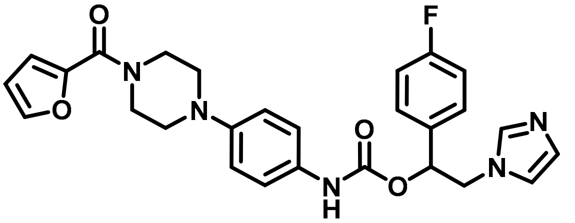	6.10	32
289	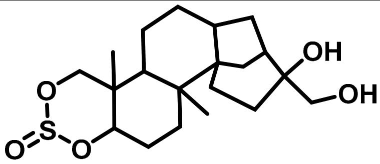	6.11	33
290	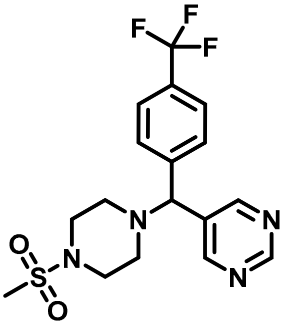	6.11	2
291	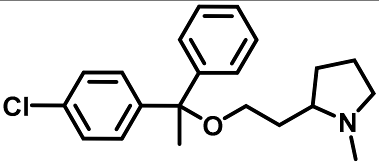	6.12	36
292*	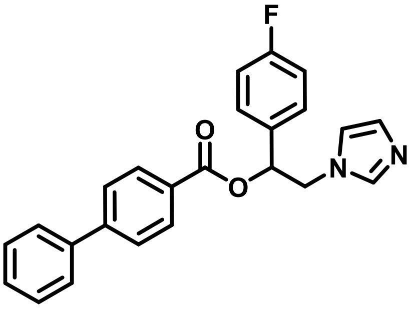	6.15	32
293	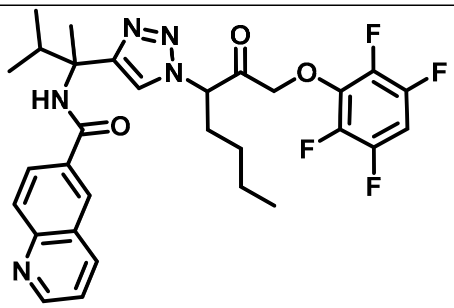	6.15	1

294	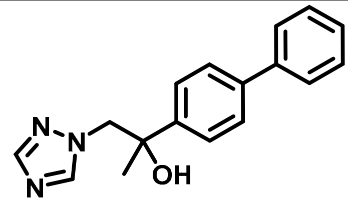	6.15	29
295	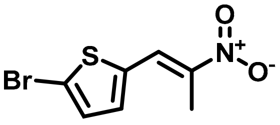	6.15	34
296*	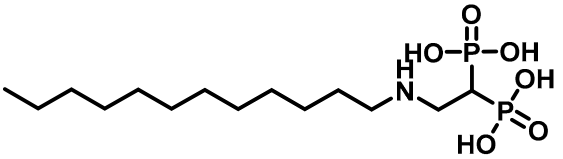	6.17	38
297	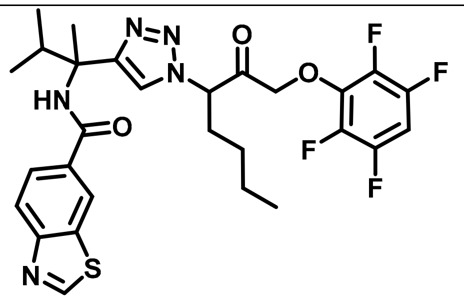	6.19	1
298	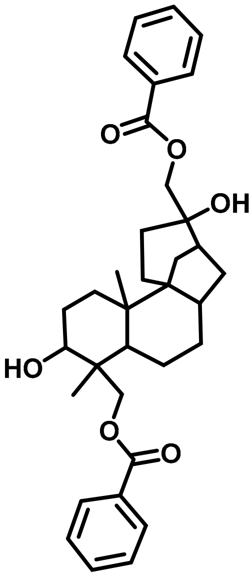	6.22	33
299	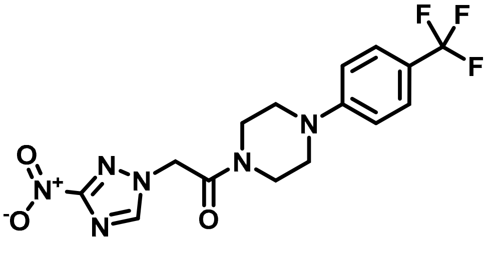	6.22	23

300	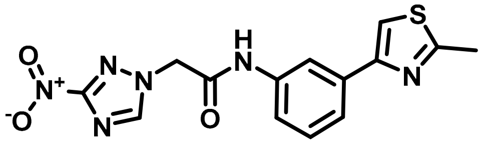	6.26	29
301*	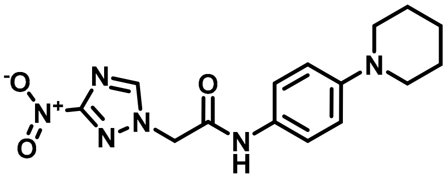	6.26	29
302	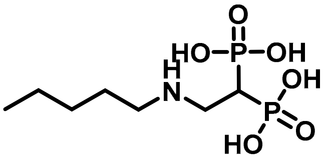	6.27	30
303	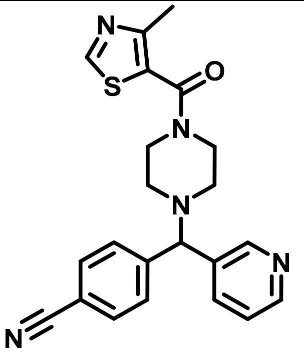	6.29	2
304	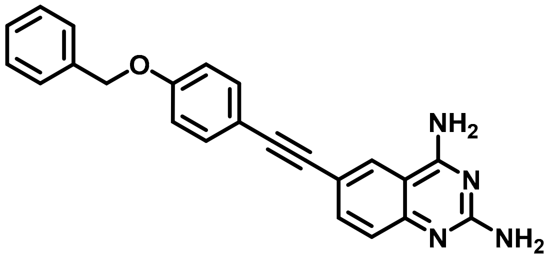	6.30	39
305	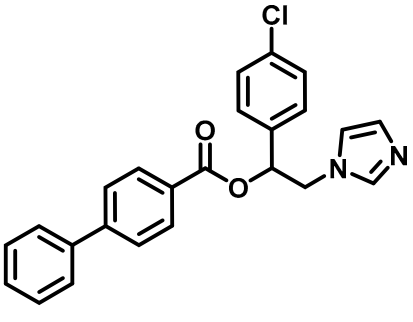	6.32	32

306	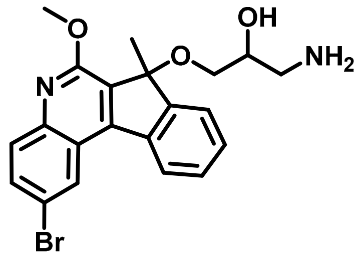	6.33	16
307*	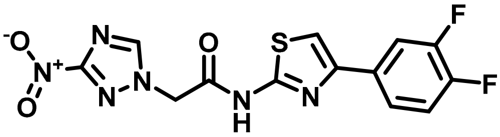	6.34	29
308	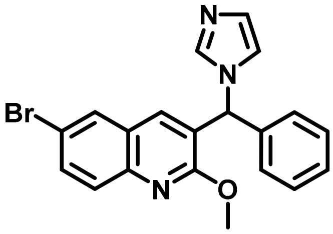	6.34	16
309*	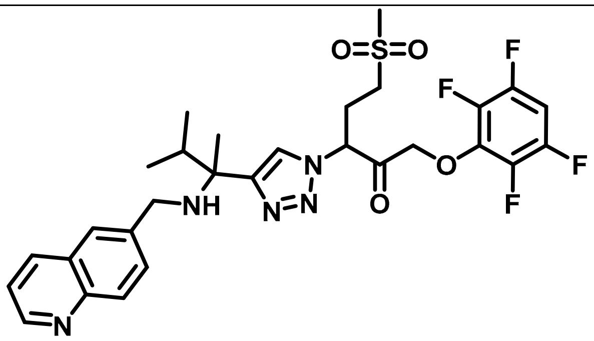	6.42	1
310	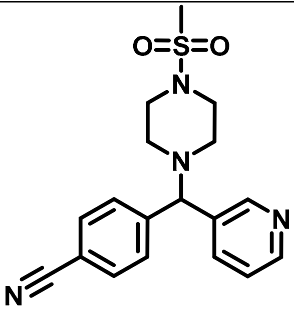	6.44	2
311	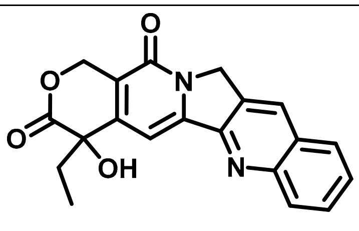	6.47	36

312	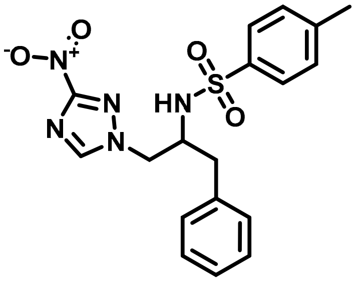	6.47	29
313*	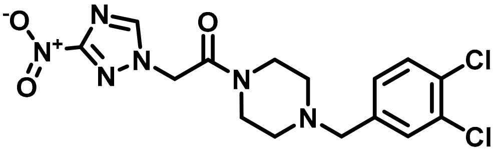	6.53	23
314	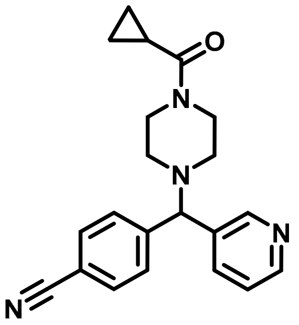	6.55	2
315	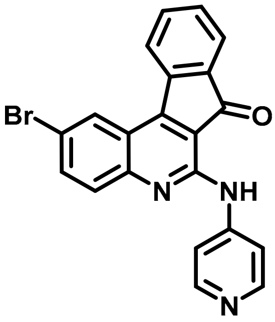	6.60	16
316	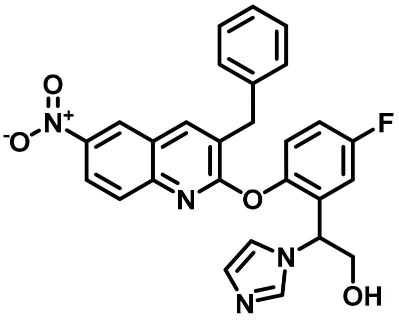	6.60	16

317*	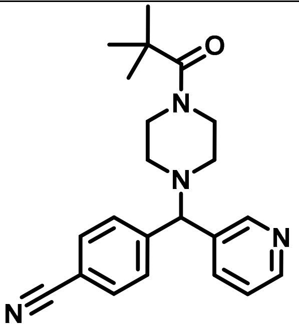	6.60	2
318	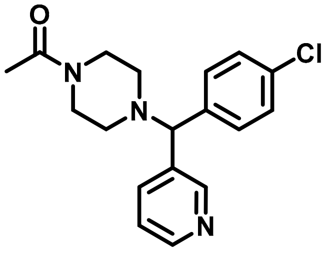	6.62	2
319	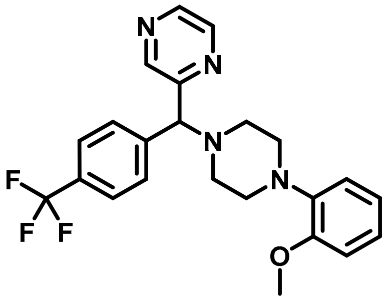	6.62	2
320	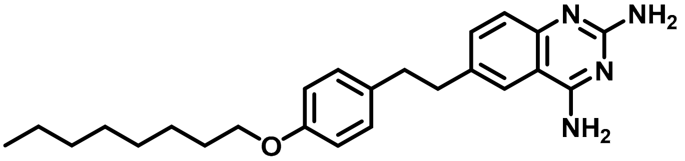	6.66	39
321*	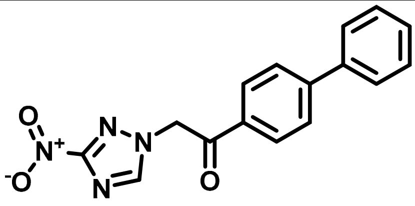	6.73	29
322	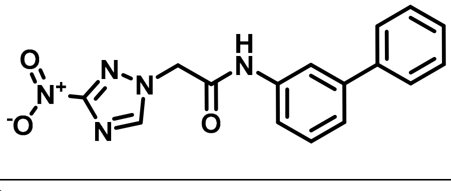	6.74	26
323	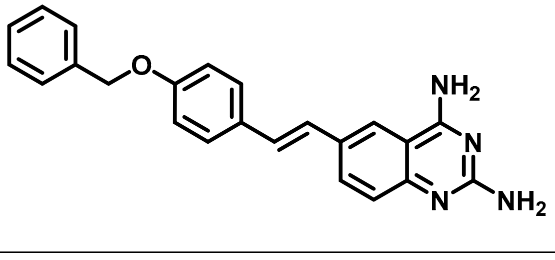	6.74	39

324	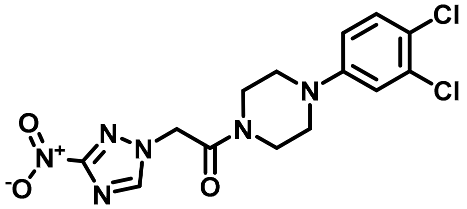	6.77	23
325	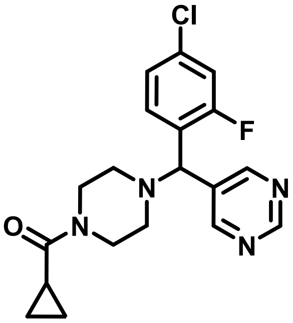	6.77	2
326*	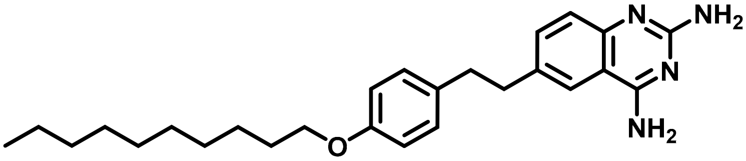	6.82	39
327	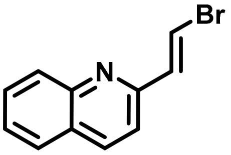	6.82	22
328	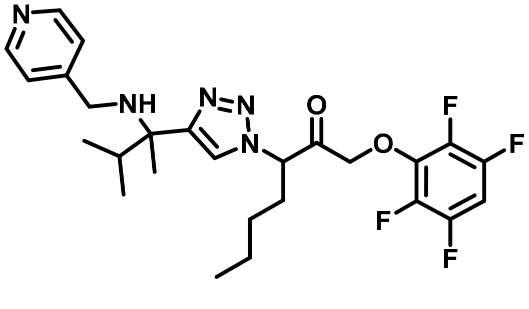	6.84	1
329	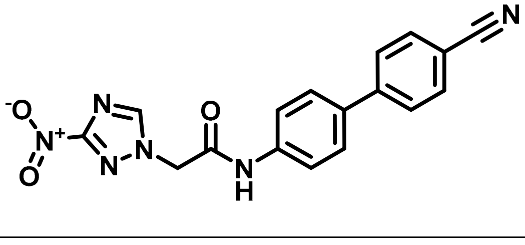	6.86	29
330	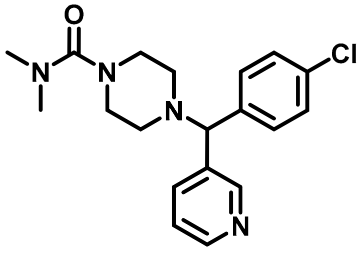	6.92	2

331*	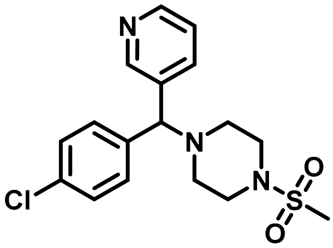	6.96	2
332	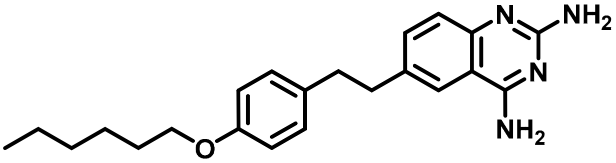	6.96	39
333	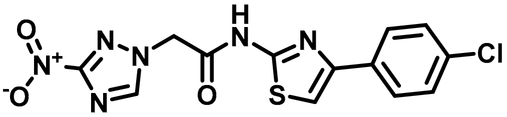	6.99	29
334	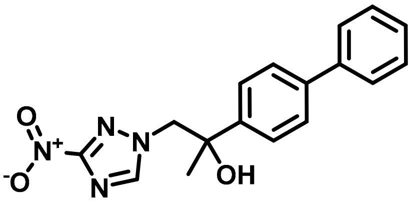	6.99	29
335	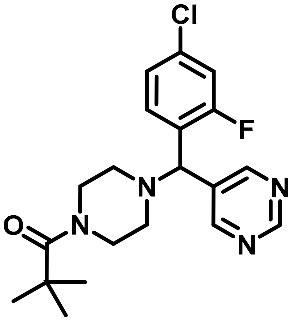	7.09	2
336	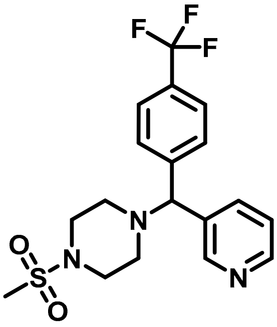	7.11	2

337*	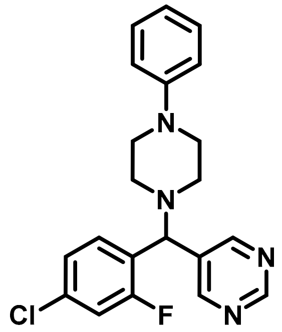	7.11	2
338	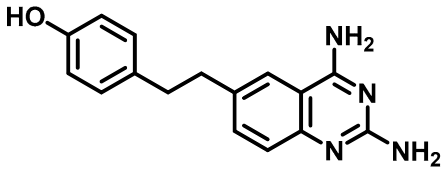	7.11	39
339	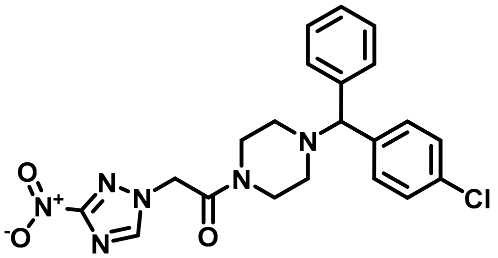	7.14	23
340	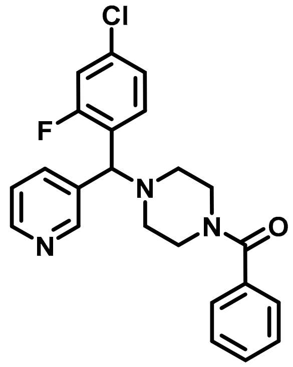	7.15	2
341*	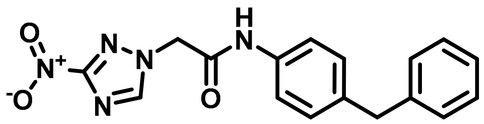	7.17	26
342	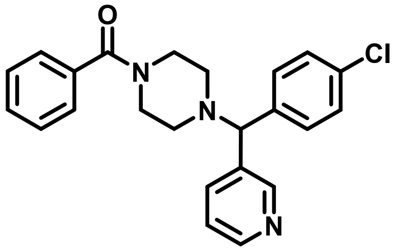	7.17	2
343**	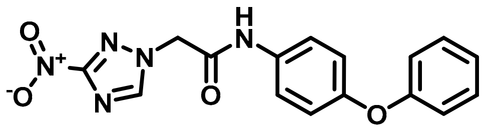	7.19	26

344**	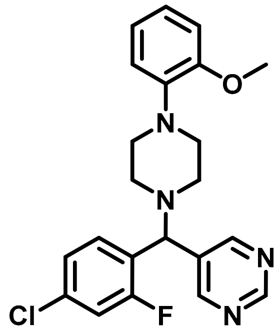	7.19	2
345**	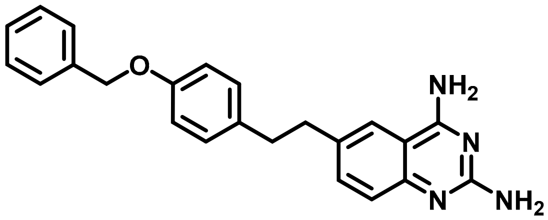	7.23	39
346*	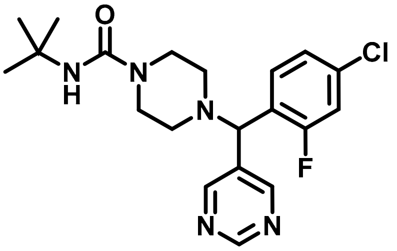	7.27	2
347	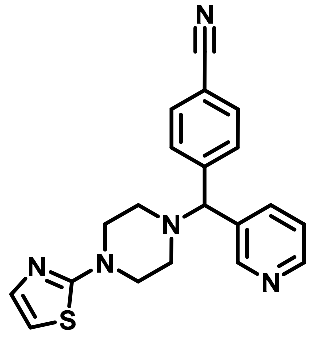	7.30	2
348*	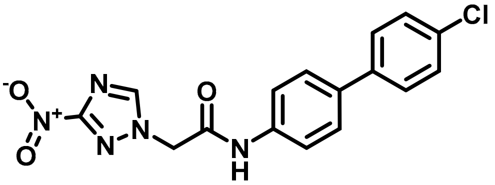	7.35	29
349**	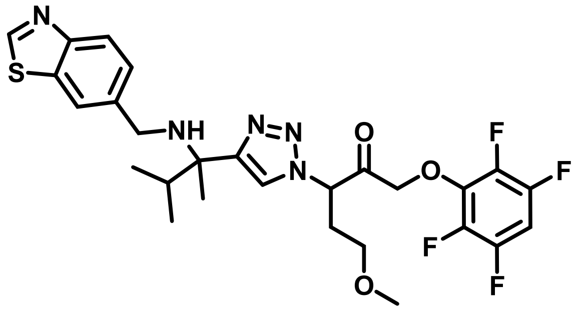	7.36	1

350**	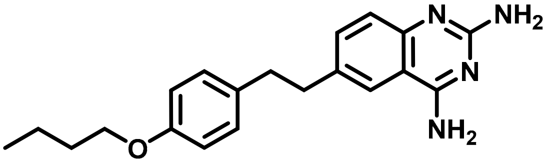	7.38	39
351	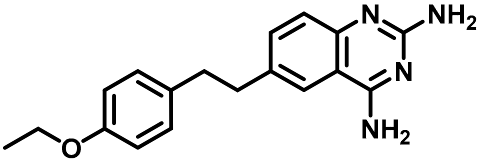	7.39	39
352**	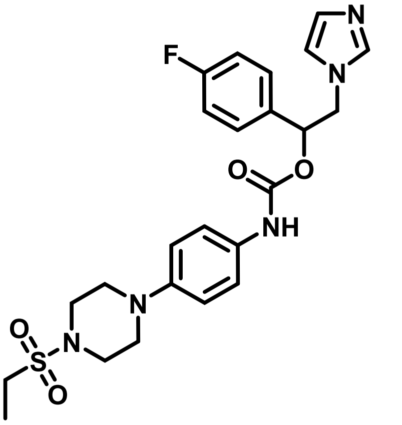	7.40	32
353*	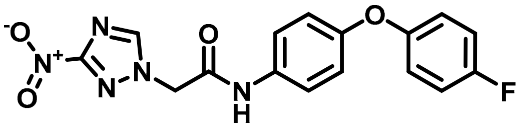	7.44	26
354**	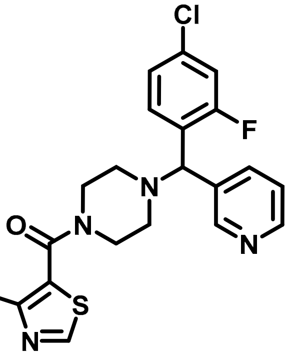	7.44	2
355	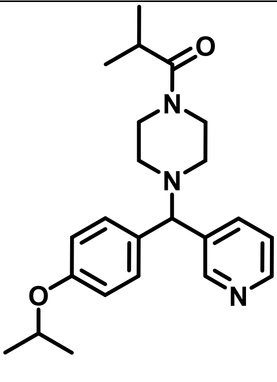	7.46	2

356	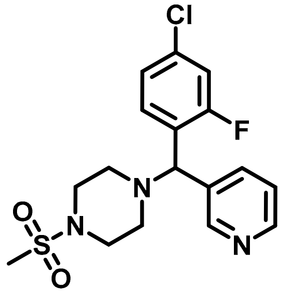	7.46	2
357	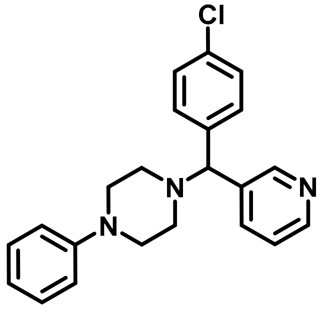	7.47	2
358*	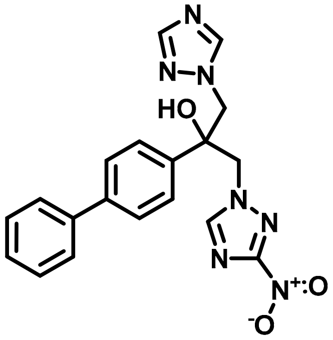	7.48	29
359	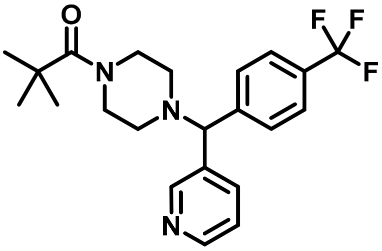	7.52	2
360**	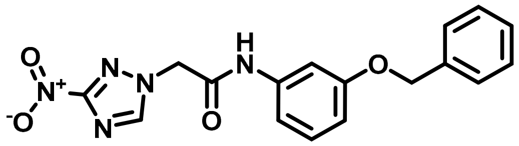	7.55	26

361**	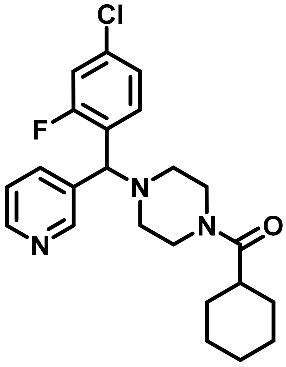	7.55	2
362*	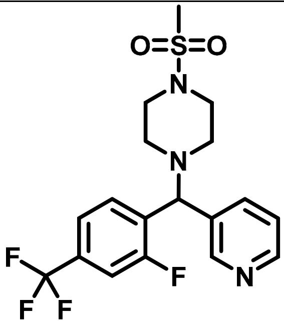	7.55	2
363	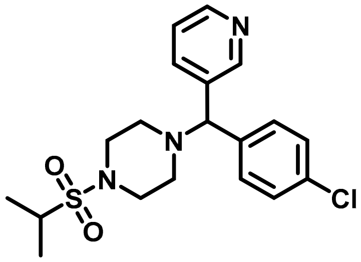	7.55	2
364	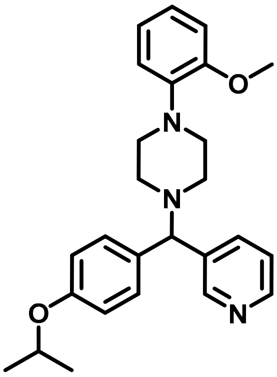	7.55	2
365	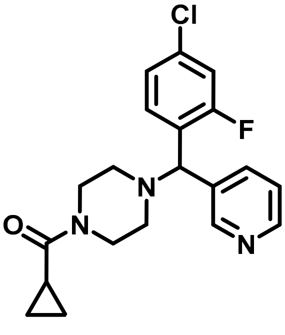	7.59	2

366*	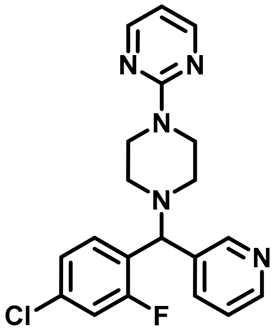	7.59	2
367	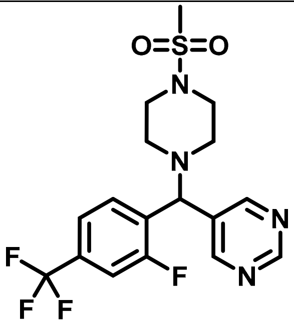	7.60	2
368	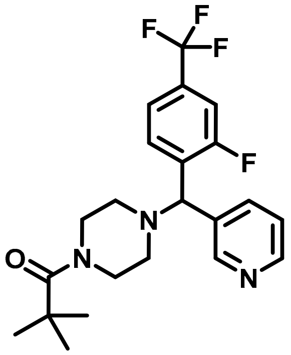	7.62	2
369	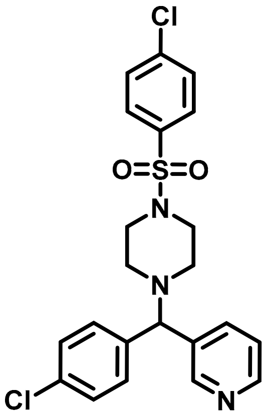	7.62	2
370**	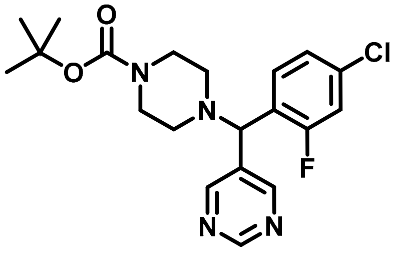	7.62	2

371*	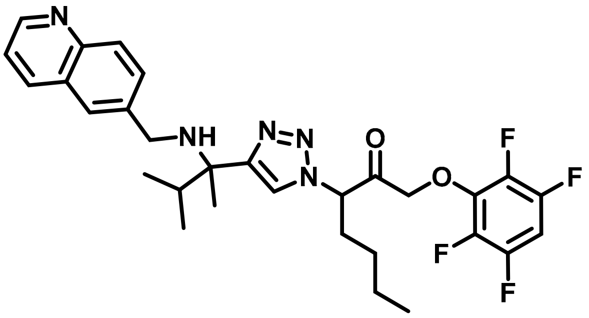	7.66	1
372	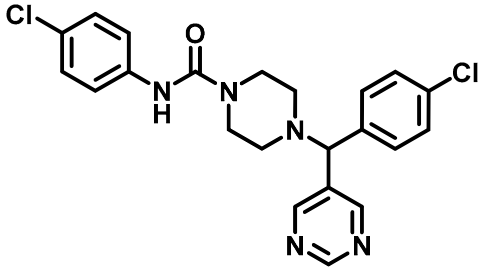	7.66	2
373**	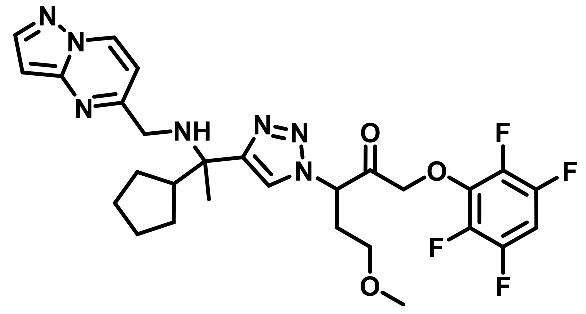	7.70	1
374	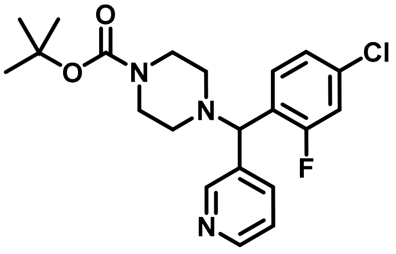	7.70	2
375	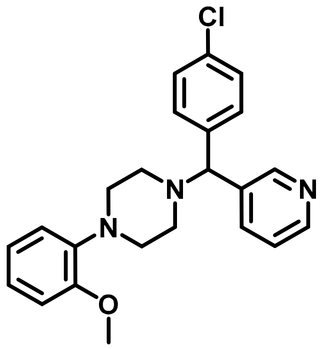	7.70	2

376*	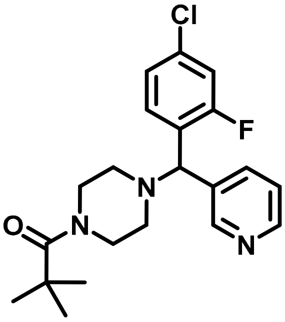	7.72	2
377**	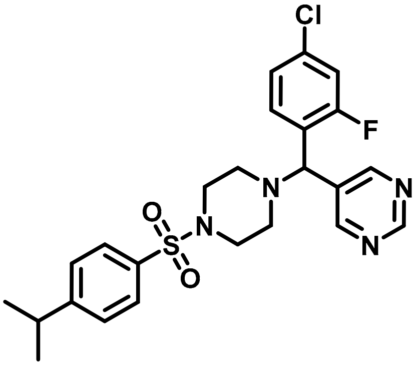	7.74	2
378**	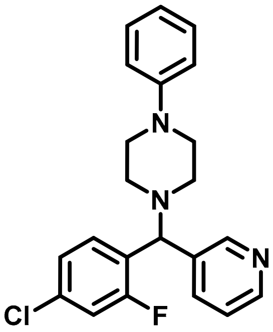	7.74	2
379**	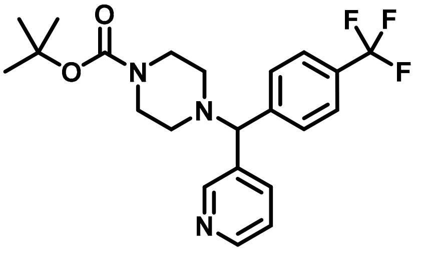	7.77	2
380**	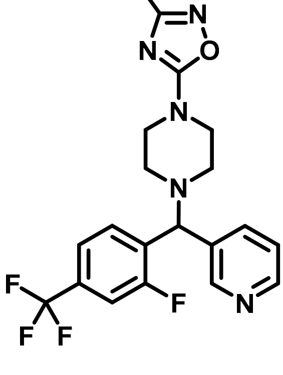	7.77	2

381*	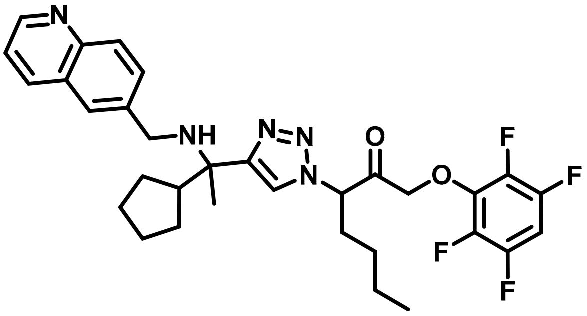	7.82	1
382	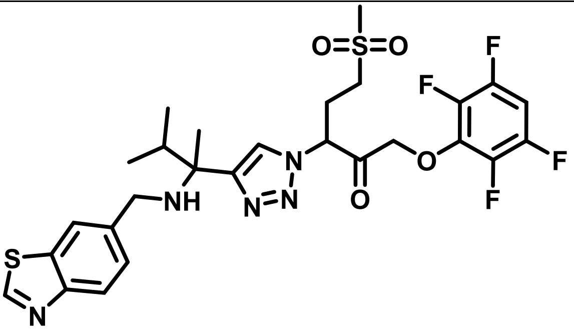	7.92	1
383**	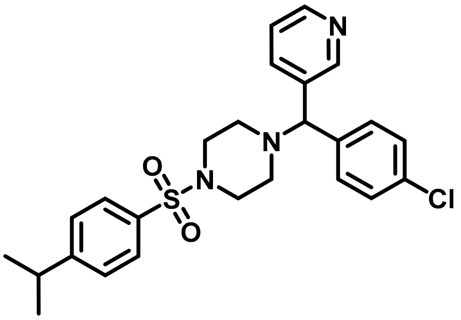	7.92	2
384	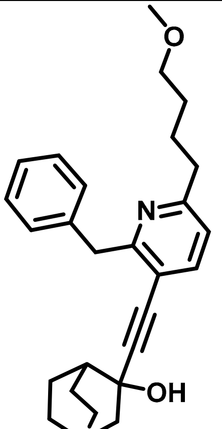	7.96	20

385**	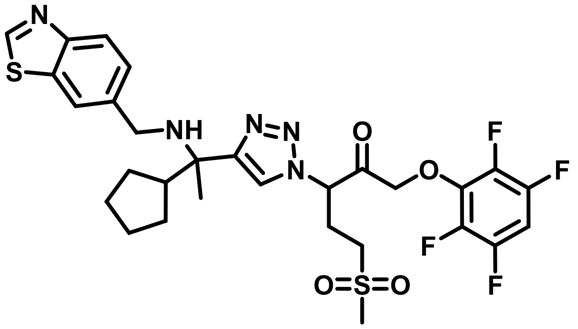	8.00	1
386*	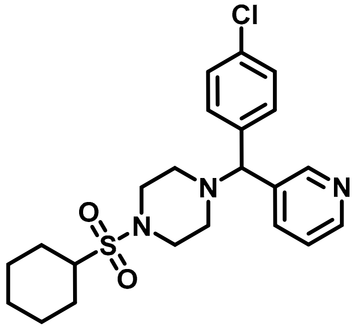	8.00	2
387	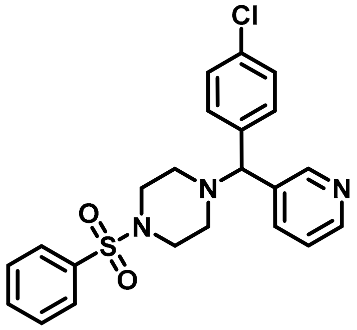	8.05	2
388**	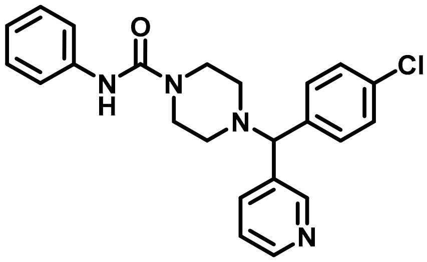	8.05	2
389*	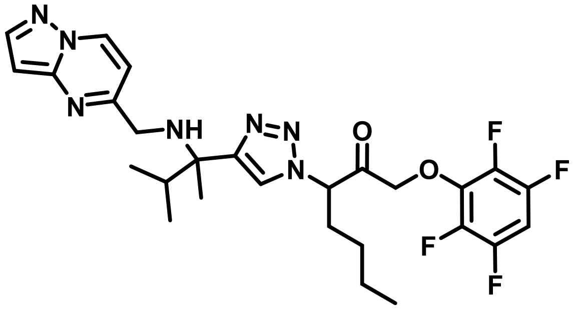	8.10	1

390**	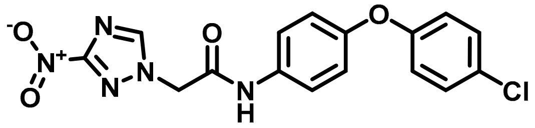	8.10	26
391**	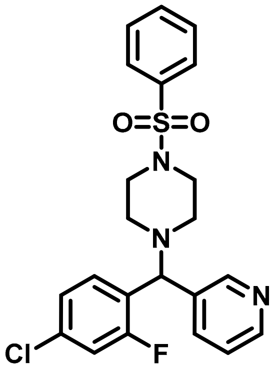	8.10	2
392**	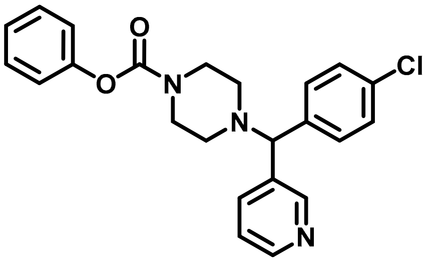	8.10	2
393**	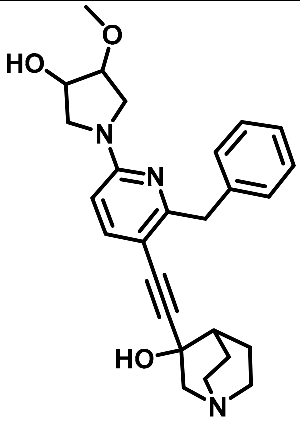	8.10	20
394*	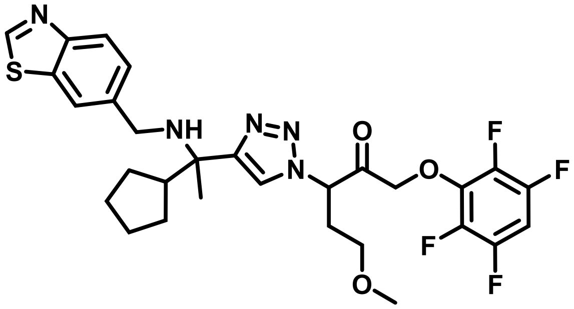	8.15	1

395**	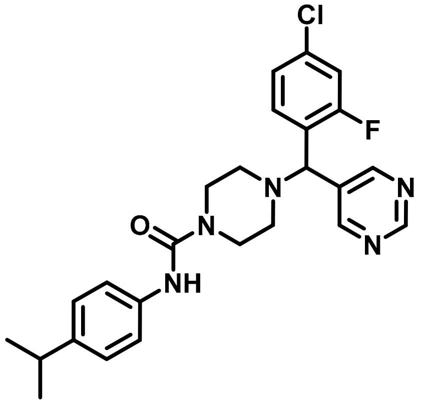	8.15	2
396**	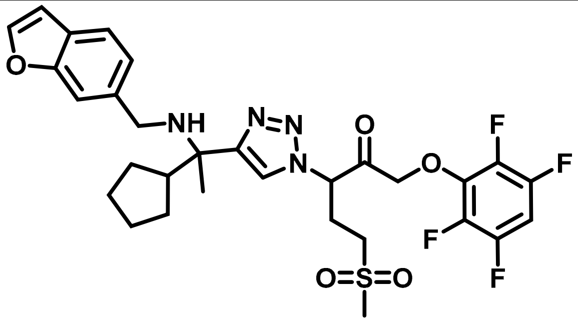	8.30	1
397*	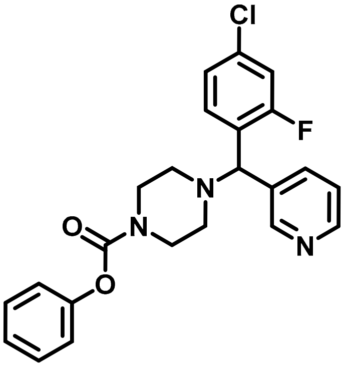	8.30	2
398*	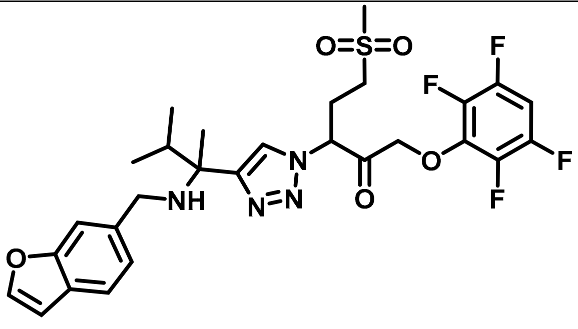	8.52	1

399**	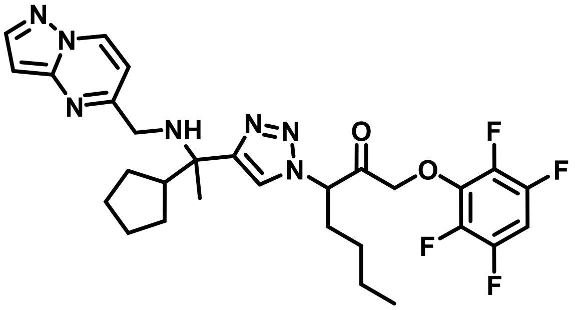	8.52	1
400*	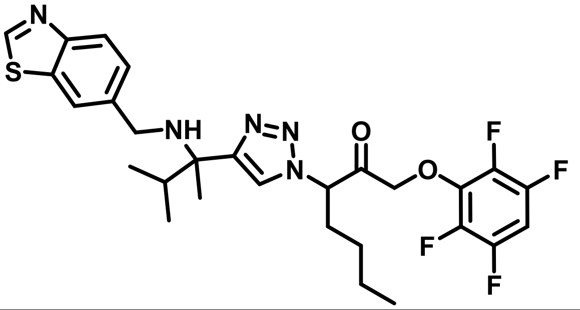	8.70	1

* Test set compounds; **Compounds used in the construction of the fragment collection.

1. Neitz, R. J.; Bryant, C.; Chen, S.; Gut, J.; Hugo Caselli, E.; Ponce, S.; Chowdhury, S.; Xu, H.; Arkin, M. R.; Ellman, J. A.; Renslo, A. R. Tetrafluorophenoxymethyl ketone cruzain inhibitors with improved pharmacokinetic properties as therapeutic leads for Chagas' disease. *Bioorganic Med. Chem. Lett.* **2015**, *25*, 4834–4837, doi:10.1016/j.bmcl.2015.06.066.
2. Keenan, M.; Alexander, P. W.; Diao, H.; Best, W. M.; Khong, A.; Kerfoot, M.; Thompson, R. C. A.; White, K. L.; Shackleford, D. M.; Ryan, E.; Gregg, A. D.; Charman, S. A.; Von Geldern, T. W.; Scandale, I.; Chatelain, E. Design, structure-activity relationship and in vivo efficacy of piperazine analogues of fenarimol as inhibitors of *Trypanosoma cruzi*. *Bioorganic Med. Chem.* **2013**, *21*, 1756–1763, doi:10.1016/j.bmc.2013.01.050.
3. Braga, S. F. P.; Alves, É. V. P.; Ferreira, R. S.; Fradico, J. R. B.; Lage, P. S.; Duarte, M. C.; Ribeiro, T. G.; Júnior, P. A. S.; Romanha, A. J.; Tonini, M. L.; Steindel, M.; Coelho, E. F.; De Oliveira, R. B. Synthesis and evaluation of the antiparasitic activity of bis-(arylmethylidene) cycloalkanones. *Eur. J. Med. Chem.* **2014**, *71*, 282–289, doi:10.1016/j.ejmech.2013.11.011.
4. Szajnman, S. H.; Ravaschino, E. L.; Docampo, R.; Rodriguez, J. B. Synthesis and biological evaluation of 1-amino-1,1-bisphosphonates derived from fatty acids against *Trypanosoma cruzi* targeting farnesyl pyrophosphate synthase. *Bioorganic Med. Chem. Lett.* **2005**, *15*, 4685–4690, doi:10.1016/j.bmcl.2005.07.060.
5. Jones, S. M.; Urch, J. E.; Kaiser, M.; Brun, R.; Harwood, J. L.; Berry, C.; Gilbert, I. H. Analogues of thiolactomycin as potential antimalarial and anti-trypanosomal agents. *J. Med. Chem.* **2005**, *48*, 5932–5941, doi:10.1021/jm049067d.
6. Gómez-Ayala, S.; Castrillón, J. A.; Palma, A.; Leal, S. M.; Escobar, P.; Bahsas, A. Synthesis, structural elucidation and in vitro antiparasitic activity against *Trypanosoma cruzi* and Leishmania chagasi parasites of novel tetrahydro-1-benzazepine derivatives. *Bioorganic Med. Chem.* **2010**, *18*, 4721–4739, doi:10.1016/j.bmc.2010.05.018.
7. da Rosa, R.; de Moraes, M. H.; Zimmermann, L. A.; Schenkel, E. P.; Steindel, M.; Bernardes, L. S. C. Design and synthesis of a new series of 3,5-disubstituted isoxazoles active against *Trypanosoma cruzi* and Leishmania amazonensis. *Eur. J. Med. Chem.* **2017**, *128*, 25–35, doi:10.1016/j.ejmech.2017.01.029.
8. Palma, A.; Yépes, A. F.; Leal, S. M.; Coronado, C. A.; Escobar, P. Synthesis and in vitro activity of new tetrahydronaphtho[1,2-b]azepine derivatives against *Trypanosoma cruzi* and Leishmania chagasi parasites. *Bioorganic Med. Chem. Lett.* **2009**, *19*, 2360–2363, doi:10.1016/j.bmcl.2008.05.013.
9. Carvalho, S. A.; Feitosa, L. O.; Soares, M.; Costa, T. E. M. M.; Henriques, M. G.; Salomão, K.; De Castro, S. L.; Kaiser, M.; Brun, R.; Wardell, J. L.; Wardell, S. M. S. V.; Trossini, G. H. G.; Andricopulo,

A. D.; Da Silva, E. F.; Fraga, C. A. M. Design and synthesis of new (E)-cinnamic N-acylhydrazones as potent antitrypanosomal agents. *Eur. J. Med. Chem.* **2012**, *54*, 512–521, doi:10.1016/j.ejmech.2012.05.041.
10. Galiana-Roselló, C.; Bilbao-Ramos, P.; Dea-Ayuela, M. A.; Rolón, M.; Vega, C.; Bolás-Fernández, F.; García-España, E.; Alfonso, J.; Coronel, C.; González-Rosende, M. E. In vitro and in vivo antileishmanial and trypanocidal studies of new N-benzene- and N-naphthalenesulfonamide derivatives. *J. Med. Chem.* **2013**, *56*, 8984–98, doi:10.1021/jm4006127.
11. Eberle, C.; Lauber, B. S.; Fankhauser, D.; Kaiser, M.; Brun, R.; Krauth-Siegel, R. L.; Diederich, F. Improved Inhibitors of Trypanothione Reductase by Combination of Motifs: Synthesis, Inhibitory Potency, Binding Mode, and Antiprotozoal Activities. *ChemMedChem* **2011**, *6*, 292–301, doi:10.1002/cmdc.201000420.
12. Bringmann, G.; Brun, R.; Kaiser, M.; Neumann, S. Synthesis and antiprotozoal activities of simplified analogs of naphthylisoquinoline alkaloids. *Eur. J. Med. Chem.* **2008**, *43*, 32–42, doi:10.1016/j.ejmech.2007.03.003.
13. King-Keller, S.; Li, M.; Smith, A.; Zheng, S.; Kaur, G.; Yang, X.; Wang, B.; Docampo, R. Chemical validation of phosphodiesterase C as a chemotherapeutic target in *Trypanosoma cruzi*, the etiological agent of Chagas' disease. *Antimicrob. Agents Chemother.* **2010**, *54*, 3738–3745, doi:10.1128/AAC.00313-10.
14. de Menezes, D. da R.; Calvet, C. M.; Rodrigues, G. C.; de Souza Pereira, M. C.; Almeida, I. R.; de Aguiar, A. P.; Supuran, C. T.; Vermelho, A. B. Hydroxamic acid derivatives: a promising scaffold for rational compound optimization in Chagas disease. *J. Enzyme Inhib. Med. Chem.* **2016**, *31*, 964–973, doi:10.3109/14756366.2015.1077330.
15. Szajnman, S. H.; Montalvetti, A.; Wang, Y.; Docampo, R.; Rodriguez, J. B. Bisphosphonates derived from fatty acids are potent inhibitors of *Trypanosoma cruzi* farnesyl pyrophosphate synthase. *Bioorganic Med. Chem. Lett.* **2003**, *13*, 3231–3235, doi:10.1016/S0960-894X(03)00663-2.
16. Upadhayaya, R. S.; Dixit, S. S.; Földesi, A.; Chattopadhyaya, J. New antiprotozoal agents: Their synthesis and biological evaluations. *Bioorganic Med. Chem. Lett.* **2013**, *23*, 2750–2758, doi:10.1016/j.bmcl.2013.02.054.
17. Magaraci, F.; Jimenez Jimenez, C.; Rodrigues, C.; Rodrigues, J. C. F.; Vianna Braga, M.; Yardley, V.; De Luca-Fradley, K.; Croft, S. L.; De Souza, W.; Ruiz-Perez, L. M.; Urbina, J.; Gonzalez Pacanowska, D.; Gilbert, I. H. Azasterols as Inhibitors of Sterol 24-Methyltransferase in Leishmania Species and *Trypanosoma cruzi*. *J. Med. Chem.* **2003**, *46*, 4714–4727, doi:10.1021/jm021114j.
18. Zuccotto, F.; Brun, R.; Gonzalez, P. D.; Ruiz, P. L. M.; Gilbert, I. H. The structure-based design and synthesis of selective inhibitors of *Trypanosoma cruzi* dihydrofolate reductase. *Bioorganic Med. Chem. Lett.* **1999**, *9*, 1463–1468.
19. Eberle, C.; Burkhard, J. A.; Stump, B.; Kaiser, M.; Brun, R.; Krauth-Siegel, R. L.; Diederich, F. Synthesis, inhibition potency, binding mode, and antiprotozoal activities of fluorescent inhibitors of trypanothione reductase based on mepacrine-conjugated diaryl sulfide scaffolds. *ChemMedChem* **2009**, *4*, 2034–2044, doi:10.1002/cmdc.200900327.
20. Sealey-Cardona, M.; Cammerer, S.; Jones, S.; Ruiz-Pérez, L. M.; Brun, R.; Gilbert, I. H.; Urbina, J. A.; González-Pacanowska, D. Kinetic characterization of squalene synthase from *Trypanosoma cruzi*: Selective inhibition by quinuclidine derivatives. *Antimicrob. Agents Chemother.* **2007**, *51*, 2123–2129, doi:10.1128/AAC.01454-06.
21. Blanco, M. C.; Escobar, P.; Leal, S. M.; Bahsas, A.; Cobo, J.; Nogueras, M.; Palma, A. Synthesis of novel polysubstituted (2SR,4RS)-2-heteroaryltetrahydro-1,4- epoxy-1-benzazepines and cis-2-heteroaryl-4-hydroxytetrahydro-1H-1-benzazepines as antiparasitic agents. *Eur. J. Med. Chem.* **2014**, *86*, 291–309, doi:10.1016/j.ejmech.2014.08.055.
22. Franck, X.; Fournet, A.; Prina, E.; Mahieux, R.; Hocquemiller, R.; Figadère, B. Biological evaluation of substituted quinolines. *Bioorganic Med. Chem. Lett.* **2004**, *14*, 3635–3638, doi:10.1016/j.bmcl.2004.05.026.
23. Papadopoulou, M. V.; Bloomer, W. D.; Rosenzweig, H. S.; O'Shea, I. P.; Wilkinson, S. R.; Kaiser, M. 3-Nitrotriazole-based piperazides as potent antitrypanosomal agents. *Eur. J. Med. Chem.* **2015**, *103*, 325–334, doi:10.1016/j.ejmech.2015.08.042.

24. Olmo, F.; Clares, M. P.; Marín, C.; González, J.; Inclán, M.; Soriano, C.; Urbanová, K.; Tejero, R.; Rosales, M. J.; Krauth-Siegel, R. L.; Sánchez-Moreno, M.; García-España, E. Synthetic single and double aza-scorpiand macrocycles act as inhibitors of the antioxidant enzymes iron superoxide dismutase and trypanothione reductase in *Trypanosoma cruzi* with promising results in a murine model. *RSC Adv.* **2014**, *4*, 65108–65120, doi:10.1039/C4RA09866H.
25. Olmo, F.; Urbanová, K.; Rosales, M. J.; Martín-Escolano, R.; Sánchez-Moreno, M.; Marín, C. An in vitro iron superoxide dismutase inhibitor decreases the parasitemia levels of *Trypanosoma cruzi* in BALB/c mouse model during acute phase. *Int. J. Parasitol. Drugs Drug Resist.* **2015**, *5*, 110–116, doi:10.1016/j.ijpddr.2015.05.002.
26. Papadopoulou, M. V.; Bloomer, W. D.; Rosenzweig, H. S.; O'Shea, I. P.; Wilkinson, S. R.; Kaiser, M.; Chatelain, E.; Ioset, J. R. Discovery of potent nitrotriazole-based antitrypanosomal agents: In vitro and in vivo evaluation. *Bioorganic Med. Chem.* **2015**, *23*, 6467–6476, doi:10.1016/j.bmc.2015.08.014.
27. Jonckers, T. H. M.; Van Miert, S.; Cimanga, K.; Bailly, C.; Colson, P.; De Pauw-Gillet, M. C.; Van den Heuvel, H.; Claeys, M.; Lemière, F.; Esmans, E. L.; Rozenski, J.; Quirijnen, L.; Maes, L.; Dommisse, R.; Lemière, G. L. F.; Vlietinck, A.; Pieters, L. Synthesis, cytotoxicity, and antiplasmodial and antitrypanosomal activity of new neocryptolepine derivatives. *J. Med. Chem.* **2002**, *45*, 3497–3508, doi:10.1021/jm011102i.
28. Guerra, A.; Gonzalez-Naranjo, P.; Campillo, N. E.; Varela, J.; Lavaggi, M. L.; Merlino, A.; Cerecetto, H.; González, M.; Gomez-Barrio, A.; Escario, J. A.; Fonseca-Berzal, C.; Yaluf, G.; Paniagua-Solis, J.; Páez, J. A. Novel Imidazo[4,5-c][1,2,6]thiadiazine 2,2-dioxides as antiproliferative *Trypanosoma cruzi* drugs: Computational screening from neural network, synthesis and in vivo biological properties. *Eur. J. Med. Chem.* **2017**, *136*, 223–234, doi:10.1016/j.ejmech.2017.04.075.
29. Papadopoulou, M. V.; Bloomer, W. D.; Lepesheva, G. I.; Rosenzweig, H. S.; Kaiser, M.; Aguilera-Venegas, B.; Wilkinson, S. R.; Chatelain, E.; Ioset, J. R. Novel 3-nitrotriazole-based amides and carbinols as bifunctional antichagasic agents. *J. Med. Chem.* **2015**, *58*, 1307–1319, doi:10.1021/jm5015742.
30. Rosso, V. S.; Szajnman, S. H.; Malayil, L.; Galizzi, M.; Moreno, S. N. J.; Docampo, R.; Rodriguez, J. B. Synthesis and biological evaluation of new 2-alkylaminoethyl-1,1- bisphosphonic acids against *Trypanosoma cruzi* and Toxoplasma gondii targeting farnesyl diphosphate synthase. *Bioorganic Med. Chem.* **2011**, *19*, 2211–2217, doi:10.1016/j.bmc.2011.02.037.
31. Silva-Júnior, E. F.; Silva, E. P. S.; França, P. H. B.; Silva, J. P. N.; Barreto, E. O.; Silva, E. B.; Ferreira, R. S.; Gatto, C. C.; Moreira, D. R. M.; Siqueira-Neto, J. L.; Mendonça-Júnior, F. J. B.; Lima, M. C. A.; Bortoluzzi, J. H.; Scotti, M. T.; Scotti, L.; Meneghetti, M. R.; Aquino, T. M.; Araújo-Júnior, J. X. Design, synthesis, molecular docking and biological evaluation of thiophen-2-iminothiazolidine derivatives for use against *Trypanosoma cruzi*. *Bioorganic Med. Chem.* **2016**, *24*, 4228–4240, doi:10.1016/j.bmc.2016.07.013.
32. De Vita, D.; Moraca, F.; Zamperini, C.; Pandolfi, F.; Di Santo, R.; Matheeussen, A.; Maes, L.; Tortorella, S.; Scipione, L. In vitro screening of 2-(1H-imidazol-1-yl)-1-phenylethanol derivatives as antiprotozoal agents and docking studies on *Trypanosoma cruzi* CYP51. *Eur. J. Med. Chem.* **2016**, *113*, 28–33, doi:10.1016/j.ejmech.2016.02.028.
33. Santos, G. B.; Krogh, R.; Magalhaes, L. G.; Andricopulo, A. D.; Pupo, M. T.; Emery, F. S. Semisynthesis of new aphidicolin derivatives with high activity against *Trypanosoma cruzi*. *Bioorganic Med. Chem. Lett.* **2016**, *26*, 1205–1208, doi:10.1016/j.bmcl.2016.01.033.
34. Herrera, C.; Vallejos, G. A.; Loaiza, R.; Zeledón, R.; Urbina, A.; Sepúlveda-Boza, S. In vitro activity of thienyl-2-nitropropene compounds against *Trypanosoma cruzi*. *Mem. Inst. Oswaldo Cruz* **2009**, *104*, 980–985, doi:10.1590/S0074-02762009000700007.
35. Sangenito, L. S.; d'Avila-Levy, C. M.; Branquinha, M. H.; Santos, A. L. S. Nelfinavir and lopinavir impair *Trypanosoma cruzi* trypomastigote infection in mammalian host cells and show anti-amastigote activity. *Int. J. Antimicrob. Agents* **2016**, *48*, 703–711, doi:10.1016/j.ijantimicag.2016.09.017.
36. Sykes, M. L.; Avery, V. M. Development and application of a sensitive, phenotypic, high-throughput image-based assay to identify compound activity against *Trypanosoma cruzi* amastigotes. *Int. J. Parasitol. Drugs Drug Resist.* **2015**, *5*, 215–228, doi:10.1016/j.ijpddr.2015.10.001.

37. de Azeredo, C. M. O.; Ávila, E. P.; Pinheiro, D. L. J.; Amarante, G. W.; Soares, M. J. Biological activity of the azlactone derivative EPA-35 against *Trypanosoma cruzi*. *FEMS Microbiol. Lett.* **2017**, *364*, 1–7, doi:10.1093/femsle/fnx020.
38. Szajnman, S. H.; García Liñares, G. E.; Li, Z. H.; Jiang, C.; Galizzi, M.; Bontempi, E. J.; Ferella, M.; Moreno, S. N. J.; Docampo, R.; Rodriguez, J. B. Synthesis and biological evaluation of 2-alkylaminoethyl-1,1-bisphosphonic acids against *Trypanosoma cruzi* and Toxoplasma gondii targeting farnesyl diphosphate synthase. *Bioorganic Med. Chem.* **2008**, *16*, 3283–3290, doi:10.1016/j.bmc.2007.12.010.
39. Khabnadideh, S.; Pez, D.; Musso, A.; Brun, R.; Ruiz Pérez, L. M.; González-Pacanowska, D.; Gilbert, I. H. Design, synthesis and evaluation of 2,4-diaminoquinazolines as inhibitors of trypanosomal and leishmanial dihydrofolate reductase. *Bioorganic Med. Chem.* **2005**, *13*, 2637–2649, doi:10.1016/j.bmc.2005.01.025.
